# Farseer-NMR: automatic treatment, analysis and plotting of large, multi-variable NMR data

**DOI:** 10.1007/s10858-018-0182-5

**Published:** 2018-05-11

**Authors:** João M. C. Teixeira, Simon P. Skinner, Miguel Arbesú, Alexander L. Breeze, Miquel Pons

**Affiliations:** 1 BioNMR Group, Inorganic and Organic Chemistry Department, University of Barcelona, Barcelona, Spain; 2 Astbury Centre for Structural Molecular Biology, Faculty of Biological Sciences, University of Leeds, Leeds, UK

**Keywords:** NMR spectroscopy, Data analysis, Intrinsically disordered proteins, Paramagnetic-NMR, Proteins, Chemical shift perturbations

## Abstract

We present Farseer-NMR (https://git.io/vAueU), a software package to treat, evaluate and combine NMR spectroscopic data from sets of protein-derived peaklists covering a range of experimental conditions. The combined advances in NMR and molecular biology enable the study of complex biomolecular systems such as flexible proteins or large multibody complexes, which display a strong and functionally relevant response to their environmental conditions, e.g. the presence of ligands, site-directed mutations, post translational modifications, molecular crowders or the chemical composition of the solution. These advances have created a growing need to analyse those systems’ responses to multiple variables. The combined analysis of NMR peaklists from large and multivariable datasets has become a new bottleneck in the NMR analysis pipeline, whereby information-rich NMR-derived parameters have to be manually generated, which can be tedious, repetitive and prone to human error, or even unfeasible for very large datasets. There is a persistent gap in the development and distribution of software focused on peaklist treatment, analysis and representation, and specifically able to handle large multivariable datasets, which are becoming more commonplace. In this regard, Farseer-NMR aims to close this longstanding gap in the automated NMR user pipeline and, altogether, reduce the time burden of analysis of large sets of peaklists from days/weeks to seconds/minutes. We have implemented some of the most common, as well as new, routines for calculation of NMR parameters and several publication-quality plotting templates to improve NMR data representation. Farseer-NMR has been written entirely in Python and its modular code base enables facile extension.

**Electronic supplementary material:**

The online version of this article (10.1007/s10858-018-0182-5) contains supplementary material, which is available to authorized users.

## Introduction

Flexible proteins, including fully disordered proteins, short disordered segments or interdomain linkers, as well as multibody macromolecular complexes, play a fundamental role in many key regulatory processes by which environmental information is sensed, processed or used to modulate complex responses. The reaction of these systems to environmental changes, which provide fundamental information on their function, can be experimentally investigated by exploring them under a large set of conditions and *evaluating* their multivariable response. In this regard, Nuclear magnetic resonance (NMR) generates atomic level data bearing structural and dynamic information. Over the last two decades, the information that can be extracted from NMR observables has continuously increased owing to the development of sophisticated NMR methods combined with molecular biology protocols for sample preparation, which together have considerably enriched the NMR spectroscopist’s toolkit. The advent of these advances places NMR in the front line as one of the most valuable techniques to investigate complex protein systems under a multitude of conditions (Kay 2016; Sormanni et al. 2017), which include the mapping of ligand binding sites and their affinities (Arai et al. 2012; Teilum et al. 2017), the presence of molecular crowders (Diniz et al. 2017), different chemical compositions of the solution (Sengupta et al. 2017) as well as site-directed mutations (Arbesú et al. 2017) and local post-translational modifications (Theillet et al. 2012) whose effects expand to the whole molecule. Such experimental design generates large and multivariable sets of NMR data. Complex datasets are also generated in the context of time-resolved NMR studies (Theillet et al. 2013; Mylona et al. 2016), and structural/dynamic analysis by paramagnetic observables (Mahawaththa et al. 2017) from different sources (Carlon et al. 2016; Schilder et al. 2016). Nonetheless, exploiting a combined use of such available NMR tools is not yet routine, mostly due to a lack of platforms that close the gap between the expertise of developers and the users’ needs, both at the acquisition and the analysis level.

Numerous NMR data analysis packages have been developed over the past 25 years (Maciejewski et al. 2017); this includes packages for specific applications e.g. relaxation analysis (Mandel et al. 1995; Orekhov et al. 1995; d’Auvergne and Gooley 2008), structure solution (Güntert et al. 1997; Herrmann et al. 2002; Rieping et al. 2007; Güntert 2009), paramagnetic NMR analysis (Pintacuda et al. 2004; Schmitz et al. 2006, 2008; John et al. 2007; Skinner et al. 2013), resonance assignment (Zimmerman et al. 1997; Jung and Zweckstetter 2004a, b; Narayanan et al. 2010), and comprehensive packages to perform a multitude of different analyses mainly focused on spectral processing and data representation, whilst also taking care of the associated bookkeeping (Johnson and Blevins 1994; Bartels et al. 1995; Delaglio et al. 1995; Keller 2003; Vranken et al. 2005; Lee et al. 2015; Skinner et al. 2016). In spite of the plethora of analysis that these packages can perform, which unarguably constitute milestone achievements in the field, we persistently find a gap on software availability dedicated to conversion of NMR observables in large sets of peaklists into correlated information-rich parameters from which biologically relevant answers can be extracted and comfortably presented to the community. This gap expands as the complexity of the systems under the scope of biomolecular NMR projects increases and the number of variables needed to characterise them also grows. Hence, it is often necessary to acquire a multitude of NMR spectra covering this whole range of variables—we term these datasets multivariable. Upon spectral analysis, the researcher faces the task of extracting structural information from correlated series of, maybe hundreds, NMR-peaklist text files which contain the NMR observables. Moreover, there are multiple ways of combining the variable-dependent information, increasing the complexity of the analysis and leading to an overload of repetitive tasks if peaklists are treated manually.

In addition to the above arguments, the nature of NMR users, and consequently NMR software users, has also evolved over the past two decades, from specialist NMR spectroscopists to structural biologists, who are not necessarily NMR experts, but use the technique routinely for data acquisition and analysis. As a result of the evolution of the biomolecular NMR user base, there is an increasing need for the development of a user-directed NMR pipeline, which focuses on the needs of the community as a whole. The interplay between NMR and other techniques for structural biology is accelerating this paradigm shift (Wassenaar et al. 2012, http://www.inext-eu.org). Moreover, we believe that such a pipeline should be open, easily extensible and accessible to anyone who wants to use and contribute to it, in compliance with the most up-to-date Open Access policies of the European Community (http://ec.europa.eu/research/openscience/index.cfm), and not closed to all except the software’s developers.

**Fig. 1:**
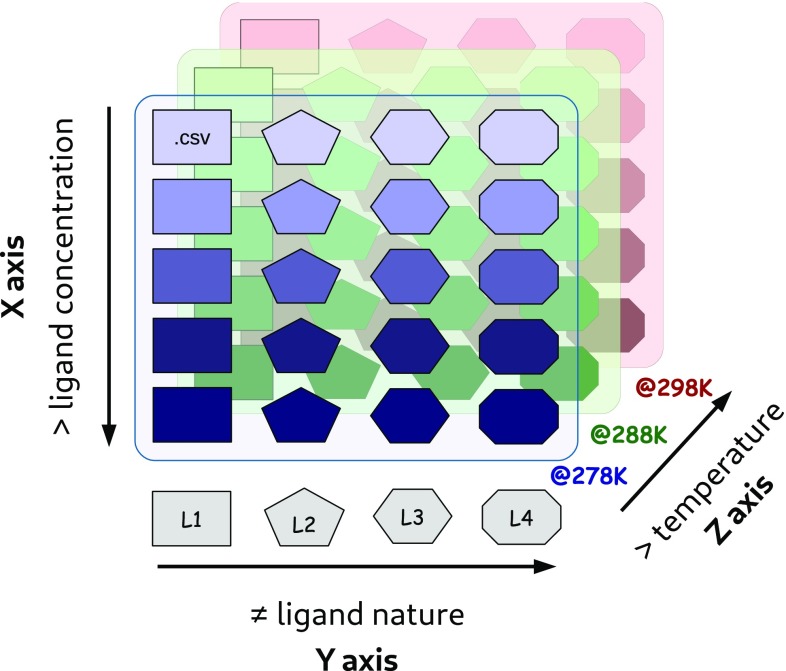
A schematic representation of the Farseer-NMR Cube. Examples of different variables are given of each Cube’s axis. Each coloured rectangle, the first one highlighted with ‘.csv’ for representation is a 2D-NMR peaklist

Taken together, the above arguments have motivated us to develop Farseer-NMR, a user-directed, open source and modular toolbox into which users can input and analyse large datasets of multivariable NMR curated peaklists in an efficient, reproducible and organized manner and without a steep learning curve. The current version has been specifically designed for the analysis of protein-related data. Farseer-NMR transforms the analysis of numerous peaklists from weeks of work to a few minutes of automatic execution, as is represented in Fig. 1 of Farseer-NMR Documentation provided in Supplementary Material, something which has been lacking in the NMR analysis pipeline. The software is coded using the Python programming language. We propose Farseer-NMR also as a nucleation point for the various NMR software packages/routines built to treat NMR-derived parameters, in particular those without a graphical user interface (GUI). We have endeavoured to facilitate this by providing a platform upon which additional routines can be developed and launched. Farseer-NMR has been developed around the concept of a “transparent box”, rather than a “black box”, or just another Python library for programmers to use. In this respect, we distribute Farseer-NMR with a complete documentation record which, together with inline code commentaries, explain the program structure and architecture, provide examples of new implementations, both for core and for the user interface, and describe the setup and execution of a Farseer-NMR calculation. All settings required for a Farseer-NMR calculation are user-settable, either using a configuration file, or via the GUI. This report describes the concepts of Farseer-NMR, its data structure and implementation of its functionalities, all of which do not require any programming knowledge.

## Methods

The Farseer-NMR Project is hosted at GitHub (https://github.com) and distributed under the GPL-3.0 license (https://www.fsf.org/); the following link directs to the project front page: https://git.io/vAueU. Farseer-NMR was written using version 3.6 of the Python programming language, and various Python libraries were used for data management and analysis. Peaklist treatment and management, parameter calculation and data fitting are performed using a combination of routines from Numpy and Scipy (van der Walt et al. 2011) and Pandas (Wes 2010), and plotting templates were developed using Matplotlib (Hunter 2007). The GUI was developed using PyQt v5.7. Version control of the code base was achieved using a git repository hosted by GitHub (http://www.github.com). Farseer-NMR was developed and tested using the GNU/Linux operating system and Anaconda Python (http://www.anaconda.com) distribution, additionally we provide Anaconda environment files for users to setup a running environment if necessary. We have also produced complete documentation containing a plethora of technical information about Farseer-NMR, its concepts and its functionalities. A maintained version of the Farseer-NMR Documentation is available online from the home page repository, the version corresponding to the current release has been supplied as Supplementary Material. At the time of this manuscript, the version of Farseer-NMR is that of an alpha-release, it is fully operational, but very much open to improvements that will be directed by the interaction with the community. The artwork in this manuscript was prepared using the Matplotlib functions developed for Farseer-NMR, the LibreOffice Suite (https://www.libreoffice.org/), Adobe Photoshop CS2 (http://www.adobe.com) and assembled with GIMP (https://www.gimp.org/).

## The Farseer-NMR structure

Farseer-NMR has been developed to enable analysis of multivariable sets of protein-related NMR data in a concerted, correlated and automated manner; variables can be continuous e.g. concentration of a titrated ligand, or discreet e.g. different protein constructs, ligands, environment. User curated peaklists from two-dimensional NMR spectra are imported into the Farseer-NMR data structure, and from here, experimental datasets can be freely navigated and exploited for analysis without any restrictions of the acquisition schedule. Up to three experimental variables can be simultaneously analysed by Farseer-NMR and, therefore, we refer to the data structure as the “Farseer-NMR cube” (Fig. 1 and Sect. II-a of the Documentation PDF), on which each dimension/axis, *x, y* or *z*, of the cube represents a different experimental variable and each cube data point, a coordinate within these axes, is an NMR peaklist table composed of rows that represent the protein residues and columns that contain all residue-related information extracted from the NMR spectra alongside user annotations. This configuration yields a final 5D matrix-like structure stored in a single digital object. The Farseer-NMR Cube can be traversed along the different dimensions describing the dependency of the data on the different defined variables.

For example, if a data set contains diamagnetic and paramagnetic spectra of two different protein constructs, into which a ligand concentration range was titrated, Farseer-NMR can analyse the spectral variations (for example, chemical shifts, intensities, linewidths, couplings) with respect to ligand concentration and for each construct separately (extracting information that can be used to calculate binding affinities, *x* axis), *calculate* the changes caused by a given ligand concentration between the two constructs (*y* axis), and extract paramagnetic relaxation enhancements (PRE) for all spectra by comparing the paramagnetic series with the diamagnetic one (*z* axis), from which distance dependent information can be extracted. Additionally, data calculated along different axes can be parsed and represented together along the other axes (*comparative analysis, see Case Study*). The selection of different dimensions of the cube for analysis and comparison across dimensions are Boolean flags in the setup of a Farseer-NMR calculation (*vide infra*). Axes can have any number of data points (peaklists) without compromising the organization and representation of results.

Any type of data can be specified along any of the different *x, y*, and *z* axes; however, for the sake of simplicity and clarity, some analysis routines are currently restricted to specific axes, e.g. paramagnetic analysis can only be performed along the *z* axis, inclusion of different protein sequences can only be performed along the *y* axis, and fitting of continuous data can only be performed along the *x* axis. Complete descriptions of axis characteristics can be found in Sect. V-d of the Farseer-NMR Documentation.

## The Farseer-NMR workflow

One of the great advantages of having all experimental data in one computational object, the Farseer-NMR Cube, is the ability to slice the cube in any direction and interrogate the data to investigate specific questions or phenomena which are not limited to the acquisition schedule. The first step of any Farseer-NMR calculation is to load the user curated peaklists as digital tables. Farseer-NMR accepts as input peaklists from a variety of formats, namely, Ansig, CCPN Analysis version 2 (Vranken et al. 2005), NMRPipe (Delaglio et al. 1995), NmrView (Johnson and Blevins 1994) and Sparky (Lee et al. 2015), taking a step towards the integration of the NMR community as a whole. Once imported, the peaklists are converted into an enhanced format initially derived from CCPN Analysis version 2 and are stored in a variable-defined hierarchical directory structure created by Farseer-NMR. The peaklists dataset is loaded to memory as a nested dictionary. At this stage, peaklists are scanned for identification of *missing* and/or *unassigned* residues along the first variable (*x* axis) and new rows representing these residues are added to the peaklists. This serves two purposes: identification of those residues and normalization of the peaklists to the same number of rows; these procedures are described in detail in Sect. II-c of the Farseer-NMR Documentation. Amide sidechain entries are also identified at this stage, if required. Following peaklist treatment, the Farseer-NMR Cube is generated.

All settings required by Farseer-NMR for a calculation: selection of dimensions for analysis, types of analysis, types of output plots and settings for the individual plot types, are specified in a JSON file (http://www.json.org/), which can be prepared manually or via the GUI (*vide infra*). Once executed, Farseer-NMR sequentially generates series of peaklists from the Farseer-NMR cube, which represent the system’s evolution along an axis/variable at fixed data points of the other two axes and walks through all the possible combinations of x, y and z variables, (Sect. II-b of the Farseer-NMR Documentation) and analyses these series according to the user-specified settings.

## Farseer-NMR analysis routines

Each series extracted from the Farseer-NMR cube can be analysed using a number of analysis routines, which are user-configurable. Some commonly used calculation and plotting routines for NMR analysis of macromolecular data have been implemented in the Farseer-NMR code base; we have also included novel methods/templates among which is the recently developed ΔPRE method (Arbesú et al. 2017). In addition, and more importantly, the core of Farseer-NMR has been designed such that virtually any calculation or plotting routine can be implemented. Farseer-NMR analysis routines consist of three steps, namely, (i) calculation of NMR parameters from raw data, (ii) data and values export and (iii) plotting. The current version of Farseer-NMR can calculate nucleus-specific and combined chemical shift perturbations (CSPs), according to Williamson (2013), height and volume ratios, and ΔPRE values according to Arbesú et al. (2017). Elaborated NMR parameters are calculated by *evaluating* each peaklist in an experimental series with the appropriate reference experiment, which is the first in the series, and the calculated values are stored in the corresponding peaklist in newly added columns. Farseer-NMR also contains a restraint fitting platform, whereby continuous data can be fit, currently restricted to data contained along the *x* axis. It is not the aim of Farseer-NMR to provide, presently, a complete suite of routines for fitting the most diverse natures of continuous NMR data, instead, to provide a platform for which users/developers can implement their own fitting routines that can plug into the core Farseer-NMR routines, and, in this way, open the door for an easy distribution among the whole NMR community.

**Fig. 2:**
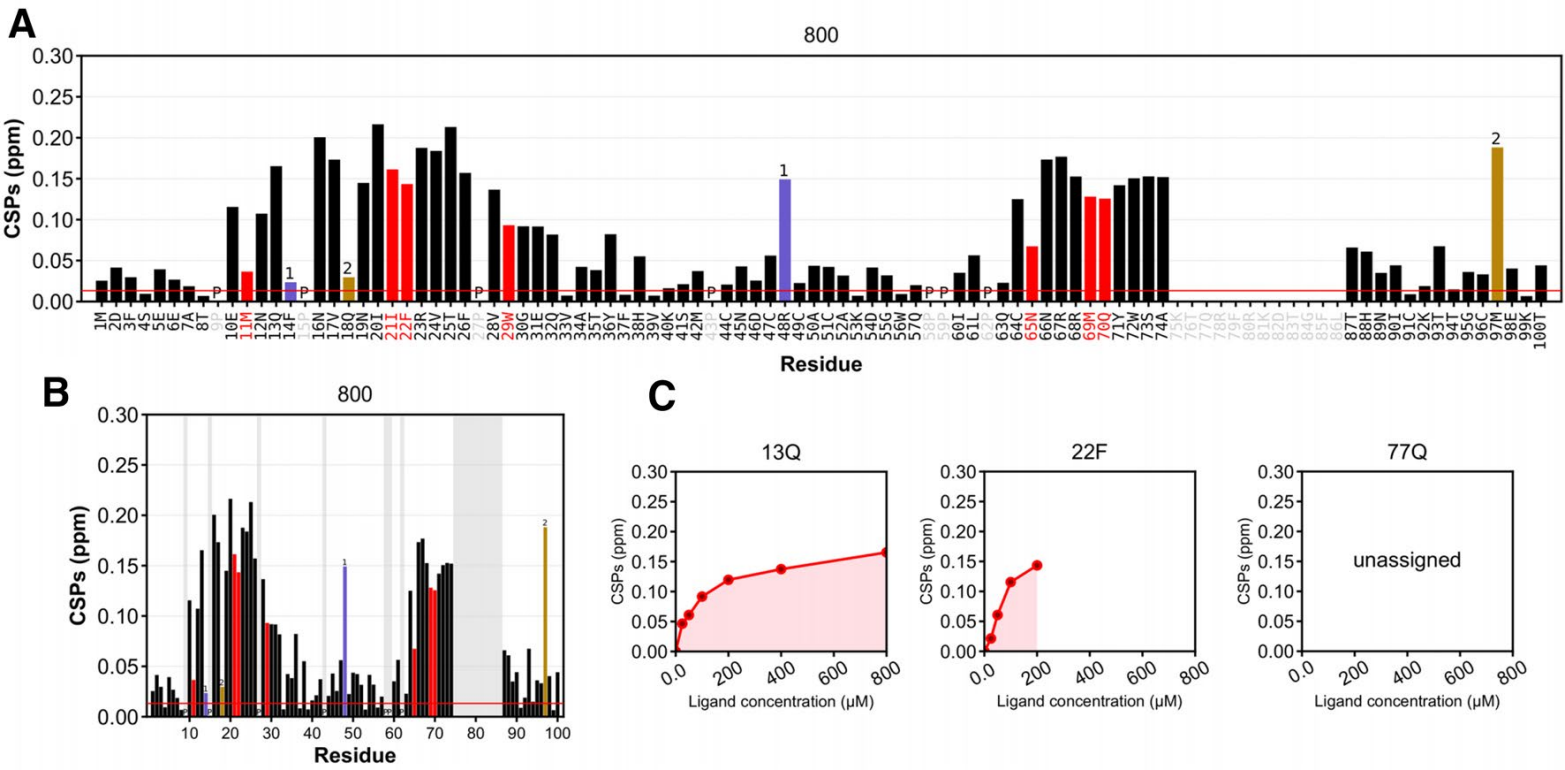
Three examples of the implemented Farseer-NMR plotting templates. ^1^H and ^15^N chemical shifts were generated from a synthetic data set simulating seven points of a ligand titration of a 100 residues protein. **a** Extended bar plot template and **b** compacted bar plot template representing the combined chemical shift perturbations (CSP), calculated according to Williamson (2013), for the last experiment in the series (with 800 µM ligand) versus the reference experiment (ligand free). Black bars represent residues measured in that spectrum. Unassigned residues are represented in grey **a** × ticks or **b** background shade. Red bars represent missing residues, i.e. residues that have been observed previously in the series but have disappeared at a given ligand concentration; the last measured value is kept. Prolines are identified by character “P”. Blue and gold bars (labelled 1 and 2, respectively) represent two pairs of residues with alternative assignment, that after plot analysis can be swapped with confidence (14F ↔ 48R, 18Q ↔ 97M), colours and labels are representations of the user annotations present in the original peaklists. A significance threshold is represented as a red line. **c** Progression of the CSP parameter represented individually for each residue. Peaks that have disappeared along the series are easily identified (22F), as well as unassigned peaks. The represented plots are crops of the full pictures generated by Farseer-NMR that represent the whole series. All colours, sizes, shapes, and labels are user-configurable

During the calculation run, a set of folders, organised hierarchically according to the defined variables, is created to store all Farseer-NMR output, so that the user can readily access the generated results. Within these folders, formatted, treated and parsed peaklists are exported as comma-separated files, which contain the newly calculated NMR parameters in addition to the original data. Moreover, the user-specified plots are created alongside parsed text tables containing only the represented data. UCSF Chimera (Pettersen et al. 2004) attribute files are generated to allow straightforward representation of the calculated restraints in 3D molecular structures; facile extension of this feature to meet other molecular representation software requirements is possible. At present, Farseer-NMR contains eight plotting templates (three bar plots, two scatter plots, a heat map and two continuous data plots), which have been prepared specifically for NMR-derived data representation. These represent some of the most common plotting styles in NMR literature, along with novel methods of presenting NMR-derived data. The plots generated by Farseer-NMR are composed of subplots that either represent data for all residues in a given series, or residue-specific variations for a given variable (where appropriate). This enables facile identification of significant results from a single figure, either by condition or by residue. Default configurations for all plots have been implemented, which yield publication-quality figures, although templates can be fully configured by the user. Figure 2 shows examples of three of the plotting templates while full information regarding Farseer-NMR plotting features is available in Sect. VI-d of the Farseer-NMR Documentation. If continuous data fitting is performed as part of a Farseer-NMR calculation run, the values obtained from fitting can be represented as a plot of restraint evolution per residue. Farseer-NMR has already been used to generate figures in published work (Bijlmakers et al. 2016; Marimon et al. 2016; Arbesú et al. 2017).

## The graphical user interface (GUI)

**Fig. 3:**
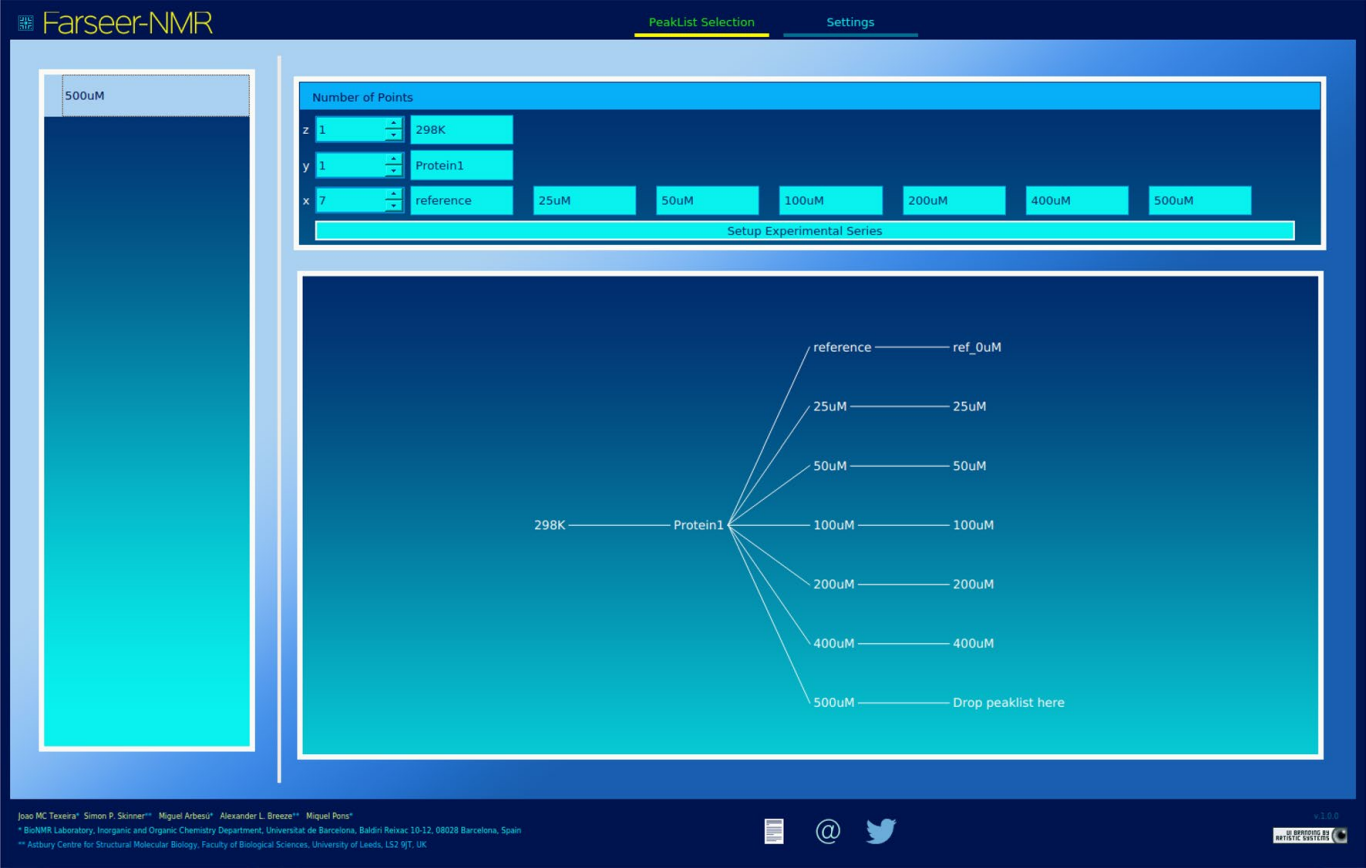
A screenshot of the Farseer-NMR user interface. The Peaklist selection tab is shown loaded with an artificial dataset as an example. The Farseer-NMR Cube variables are set up and the corresponding data points are populated with peaklists

To facilitate easy preparation and execution of Farseer-NMR calculation runs, a user-friendly GUI has been developed; full description can be found in Sect. III of the Farseer-NMR Documentation. The current GUI is divided into two tabs, namely, Peaklist Selection (Fig. 3) and Settings (Sect. III-c of the Documentation PDF). In similarity with the core code, the GUI was developed to be easily expansible and designed so that new features can be added as new tabs with their respective options and functions. We envisage user-developer contributions playing a significant role in this evolution of the interface. The Peaklist Selection tab consists of three distinct areas: a sidebar for data import and selection, an area for specifying experimental conditions, and an area for construction of the Farseer-NMR data structure. Peaklists are imported by dropping them on to the side bar, which will detect the peaklist format and import the data if the peaklist format is recognised (*vide supra*). Setting up the Farseer-NMR data structure consists of two stages: (1) specifying the numbers of points and labels for each of the axes of the Farseer-NMR cube, that is, experimental variables and number of measured data points, respectively, and (2) selection of the appropriate peaklist for each combination of variables. Once the numbers of points, and labels for each axis point have been specified, a hierarchical tree diagram representing all specified conditions is drawn with “Drop peaklist here”, written at the end of each branch. From here, peaklists corresponding to the correct combination of variables can be dragged and dropped onto the end of each branch of the tree, thereby creating the Farseer-NMR data structure.

The Settings tab of the GUI contains input fields for all the parameters needed to analyse the data using Farseer-NMR. There are two text input boxes, one for setting the *Peaklist Dataset Folder* from which peaklists can be imported aside from the drag-and-drop area, and a second for setting the Calculation Output Path to which all treated peaklists and plots will be written. Selection of axes along which to perform analysis, and options to compare across dimensions, consider side chain amide peaks *and identification* of missing and unassigned residues, are specified by checkboxes in this tab. Settings for chemical shift normalisation, NMR observable selection and parameter calculation, plotting template selection, figure format and file dimension and resolution are also configurable from this tab. A series of popups accessible from this tab contain all the settings for all the implemented plotting templates. The GUI has been forged to facilitate, and invite, advanced users to implement new calculation routines and design the appropriate setting menus under a new tab. Default settings for all of the fields, are set at start-up, and configuration files can also be loaded and saved from this tab. The footer of the GUI enables instant access to Farseer-NMR documentation, to the Farseer-NMR Twitter account, and to contact the developers via e-mail to submit feedback, request features and report any bugs.

## Case study: ΔPRE analysis

An example of the versatility of Farseer-NMR is the implementation of a new procedure for the analysis of intramolecular PRE induced by a paramagnetic probe on disordered protein fragments of a series of proteins harboring mutations that may affect their dynamics and conformational properties (Arbesú et al 2017). The data set was formed of 10 sequence variants that probed the effect of four point mutations at conserved aromatic residues and the presence of the folded domain adjacent to the disordered region by measuring the relaxation induced by paramagnetic centre incorporated in three alternative sites along the sequence. Experimental data consisted of HSQC spectra of the paramagnetic proteins and the corresponding diamagnetic control (20 experiments). The observed PREs were compared with the values expected from a random coil model for the disordered region, independently calculated using Flexible-Meccano (Ozenne et al. 2012) but incorporated into Farseer-NMR as additional (synthetic) data. Farseer-NMR generated plots showed a direct comparison of predicted and measured PRE, smoothed difference profiles obtained using a running Gaussian convolution filter, heat-map representation of the ΔPRE data, side by side comparisons, and per-residue chemical shift perturbation mapping of the whole constructs. This analysis clearly identified departures from the random coil model and the role of the conserved aromatic residues in the formation of a fuzzy complex in the N-terminal region of the Src protein.

**Fig. 4:**
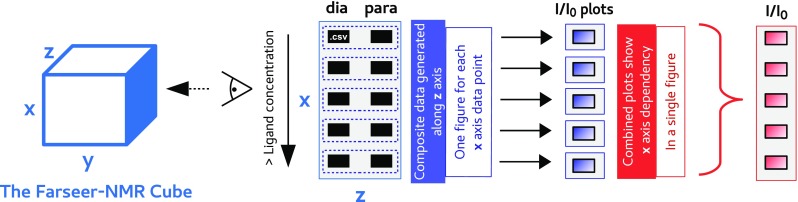
A schematic representation of the *Comparative*/*Stacking* Analysis workflow. Farseer-NMR can easily generate combined data from experiments (e.g. Intensity ratios for each residue between paramagnetic and diamagnetic samples in PRE experiments—generated along the *z* axis—and compare the derived results as a function of an external variable (e.g. ligand concentration, corresponding to the *x* axis of the Farseer-NMR’s Cube) as a single combined plot or as a PRE versus ligand concentration curves for individual residues

Technically, analysis of paramagnetic NMR data consists in representing the evolution of a paramagnetically generated NMR parameter, in this case the ΔPRE calculated along the *z* axis, along another axis that describes the system as a function of an external variable (sequence variants, point mutations, probe position). Farseer-NMR implements this algorithm under the name “*Comparative*/*Stacking Analysis*”. It serves as the basis for analysing paramagnetic NMR data as well as a parsing routine to regroup results for improved visualization. Figure 4 schematises the workflow of the Comparative Analysis, described in detail in Sect. II-d of the Farseer-NMR Documentation.

## Concluding remarks

Farseer-NMR is a software package to streamline and standardize the analysis of NMR data obtained from sets of samples exploring multiple variations in terms of protein sequence or experimental conditions altering the chemical nature of the sample, including ligand binding, or its spectroscopic properties (e.g. paramagnetic and diamagnetic forms or isotropic *versu*s oriented samples). Farseer-NMR facilitates the analysis of multivariable data by allowing the extraction of the individual contribution of the variables, and the identification and characterization of correlated effects and directly produces publication-quality plots, reducing weeks of tedious and error-prone manual analysis to a few minutes. Farseer-NMR closes a longstanding gap in the quest for creating an automated BioNMR pipeline. It is important to consider also that BioNMR-related peaklists are often analysed iteratively, that is, after restraint calculation and representation, further inspection of the spectra is often necessary to correct for interpretational errors, which may only be evident after considerable analysis. Farseer-NMR drastically reduces the time required in this process by enabling iterative analysis of a complete dataset with a single mouse “click”, using previously saved settings, which can be reloaded *a posteriori*. Farseer-NMR was developed focusing on a “box”-like concept, that is, *input → routine → output*, where routines can be as many as imaginable. Rather than being a “black box”, Farseer-NMR is an open-source “transparent box” where routines can be inspected, improved and implemented by the users themselves, enthusiast developers or via user-developer interactions. In this vein, Farseer-NMR has been designed to be user-friendly to NMR users with biological background, but also as flexible as possible to engage advanced NMR users. The software is written in Python 3.6 related libraries and is stored in a GitHub repository, which is open-source and free-to-use according to the terms of the GPL-3.0 license. We invite users to fork the repository and create pull requests to contribute to the Farseer-NMR project. Indeed, we have developed Farseer-NMR with the scope of creating a nucleation point for specialized NMR analysis routines that exist throughout the NMR community so that those can become available to the community as a whole in a easy and convenient manner.

## Electronic supplementary material

Below is the link to the electronic supplementary material.

Supplementary material 1 (PDF 12491 KB)
