# Supplementary material for: Farseer-NMR: automatic treatment, analysis and plotting of large, multi-variable NMR data
Source: J Biomol NMR. 2018 May 11;71(1):1–9. doi: 10.1007/s10858-018-0182-5 (PMC5986830; doi:10.1007/s10858-018-0182-5)
Supplement: Supplementary file 1 — Supplementary material 1 (PDF 12491 KB) [file 10858_2018_182_MOESM1_ESM.pdf]

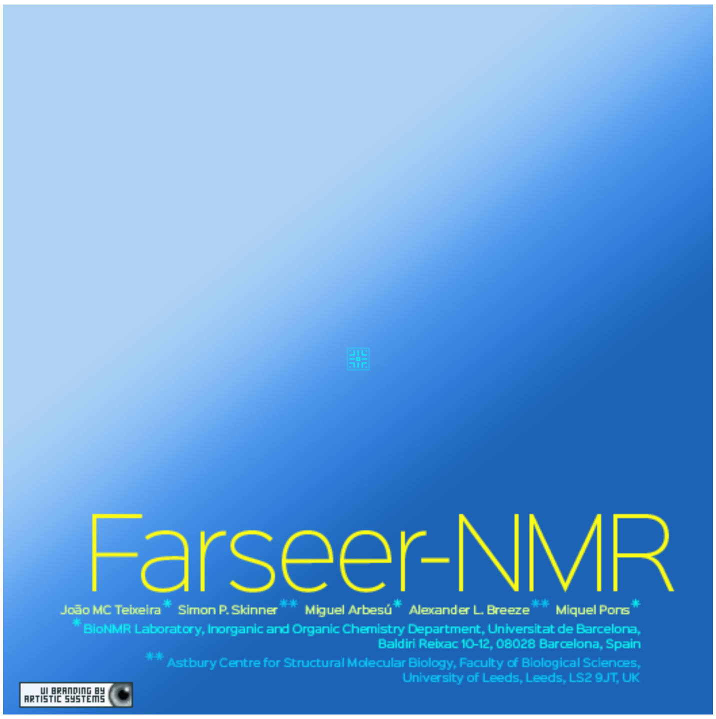

Documentation

Aim and resume

Farseer-NMR was developed to improve and facilitate the analysis of BioMolecular NMR data derived from protein analysis. Taking a series of 2D-NMR peaklists, automatic analysis, calculation and representation of the results as publication-quality plots is possible thanks to Farseer-NMR. You won't need to spend several days of meticulous work in spreadsheets or omit part of your results due to complexity. Farseer-NMR presents a straightforward manner to extract the most out of your data.

This documentation PDF is intended to explain how Farseer-NMR works conceptually, the technical details on how to set a calculation running and how to read and analyse the generated results **(which may be numerous!)**.

Table of Contents

I. Introduction and README................................7

a) What is Farseer-NMR?...7

The daily problem..7

The solution...7

How?...7

What else?..7

b) README...8

Project Repository and Download.............................8

Installation..8

1) Setting up a Miniconda for Farseer-NMR....................8

2) Creating a dedicated Anaconda environment..............8

3) Installation for advanced users...................................8

Running Farseer-NMR...9

Running without the GUI..9

Warnings, Errors and Troubleshooting (WET) list......9

Participate in the Farseer-NMR community...............9

Reporting a bug...9

Suggesting Features..9

Become a collaborator...9

Mailing list...9

Social Network..9

License...9

II. The Farseer-NMR data structure...................10

Example 1:..10

a) The Farseer-NMR Cube......................................11

b) Multidimensional Analysis Workflow.................12

A Series of NMR experiments...................................13

Navigating the Farseer-NMR Cube's Axes................14

Evolution along the X axis...15

Evolution along the Y axis...16

Evolution along the Z axis...17

Analysis of Experimental Series...............................18

c) The Farseer-NMR Analysis Routines.................19

Treating and Formatting peaklists...........................20

Technical consideration..20

Reading assignment information....................................21

Identifying missing peaks..22

Identifying unassigned residues......................................23

Performing Calculations...24

Plotting the results..25

d) Comparative/stacking Analysis..........................26

Example 2:..26

Calculating along X and stacking along Y.......................27

Calculating along Y and stacking along X.......................28

Calculating along Z and stacking along X.......................29

Stacking for each dimension/experimental variable..30

III. The Farseer-NMR Program..........................31

a) The initial input structure...................................32

Example 3:..32

b) Setting up the Experimental Tree.......................33

Logical order of the experiments..............................33

c) Settings Tab...35

d) Running without the GUI...................................36

IV. NMR Parameters...37

a) Chemical shift Differences.................................37

b) Combined Chemical Shift Perturbations (CSP)..38

c) Intensity Ratios..39

d) PRE and ΔPRE Analysis......................................40

Theoretical PRE profiles...40

ΔPRE Analysis..40

V. Other routines and Technical Details.............41

a) Compatible Peaklist Formats.............................42

b) Load/Save and the Configuration File JSON......43

c) Searching for missing residues along Y and Z....44

d) Axes restrictions...45

Along the X axis – Data fitting.................................46

Along the Y axis – Different sample constructs.........47

Along the Z axis – Paramagnetic NMR Analysis.......48

e) The FASTA file..49

How to input FASTA files...49

When running without the GUI......................................49

Practical considerations...49

f) Chemical shift normalisation..............................50

g) Theoretical PRE profiles.....................................51

When running without the GUI......................................51

h) Data Fitting...52

How to setup a Fitting..52

Generated files..52

Implementing new functions....................................52

i) Sidechain Analysis...53

VI. Output and Data organization.......................54

a) The log file..54

b) The results folder hierarchy...............................55

The Calculation Output Folder..................................55

The Backbone and Sidechains Folders......................56

The Calculations folder...56

The axes subfolders...56

Folders index prefix..56

The different series...56

Some examples:..56

The Comparisons Folder...58

Example 4:..58

c) Reading the results..59

FullPeaklists folder 59

TablesAndPlots subfolder 59

ChimeraAttributeFiles folder 59

d) Plotting Templates..60

Bar Plots..61

Compacted Bar Plot...62

Extended Bar Plot..63

Vertical Bar Plot...64

Residue Evolution Plots...65

Restraint Evolution...65

Chemical Shift Scatter Plot...66

Chemical Shift Scatter Flower Plot....67
The ΔPRE map....68
The ΔPRE heatmap....69

VII. Tutorials....70

a) Tutorial 1 – Single variable datasets....71
Context....71
Aim....71
How to....71
Results....72

b) Tutorial 2 – Analysing 2D Datasets....77
Context....77
Aim....77
How to....77
Results....78

c) Tutorial 3 – paramagnetic NMR analysis....81
Context....81
Aim....81
How To....81
Results....82

Figure Index

Figure 1: The BioNMR project pipeline. Farseer-NMR acts upon an old and persistent gap: the transformation of peaklists into human-readable and biological relevant data in the form of plots or parsed tables............................7
Figure 2: The schematic representation of the Farseer-NMR Cube...11
Figure 3: Representation of how the X axis series are generated along the Farseer-NMR Cube.............................15
Figure 4: Representation of how the Y axis series are generated along the Farseer-NMR Cube.............................16
Figure 5: Representation of how the Z axis series are generated along the Farseer-NMR Cube.............................17
Figure 6: The schematic representation of Farseer-NMR workflow and analysis routines....................................19
Figure 7: Expansion of Assign F1 column to three new assignment informative columns......................................21
Figure 8: New rows are added to identify the peaks that were missing with respect to the reference experiment. 22
Figure 9: New rows are added to identify those residues that are not assigned in the reference, and in consequence, in the whole series..23
Figure 10: An example of two experimental series that represent the evolution of P as a function of the concentration (X axis) of L1 and L2 (Y axes)..26
Figure 11: Schematic representation of how results generated along the X axis are compared along the Y axis.....27
Figure 12: Schematic representation of how results generated along the Y axis are compared along the X axis.....28
Figure 13: Schematic representation of how results generated along the Z axis are compared along the X axis.....29
Figure 14: Cropped region of the Settings tab. Selecting the folder containing the peaklist files in the via Peaklist Dataset Folder button...34
Figure 15: Adding peaklists to the Side bar, configuring the tree and populating it by drag and drop in PeakLists Selection tab...34
Figure 16: Farseer-NMR Settings tab...35
Figure 17: CSP Specific submenu...38
Figure 18: Alpha value by residue type popup menu. Here you can configure the exact alpha value for each residue type...38
Figure 19: An example of how results for the different dimensions are organised under the Farseer-NMR data structure...57
Figure 20: An example of how comparative analyses are organized under the Farseer-NMR data structure.........58
Figure 21: The folders where results from the Farseer-NMR Analysis routines are stored....................................59
Figure 22: Compacted Bar template subplot..62
Figure 23: Full picture of a 3×3 subplot table representing the CSPs evolution of the whole series........................62
Figure 24: Extended Bar template subplot...63
Figure 25: Full picture of a 7×1 subplot table representing the CSPs evolution along the whole series...................63
Figure 26: Vertical Bar template subplot..64
Figure 27: Full picture of a 5×2 subplot table representing the CSPs evolution along the whole series...................64
Figure 28: Subplot template of the restraint evolution representation per residue...65
Figure 29: The full figure as generated by Farseer-NMR where a subplot is drawn for each residue......................65
Figure 30: A subplot template representing the chemical shift perturbation of a specific peak where the reference is centred at 0.0..66
Figure 31: The full figure as generated by Farseer-NMR where a subplot is drawn for each residue......................66
Figure 32: The Chemical Shift Scatter Flower plot...67
Figure 33: ΔPRE map. In black the values for the reference experiment. In gold the values for A27C_D20 construct. Regions 0-20 (Met1 is considered Met0 in this construct) and 35-45 are shaded to grey and residues 43, 50, 56, 61 and 67 are highlighted as examples. Data from Arbesú et al. 2017..68
Figure 34: The ΔPRE heatmap templates. A) with raw data. B) with Gaussian smoothed data. Tag position if represented by a red line...69
Figure 35: Experimental Tree defined and peaklists loaded to Sidebar – Tutorial 1..74
Figure 36: Experimental Tree populated – Tutorial 1...74
Figure 37: Configurations in Settings tab for Tutorial 1...75
Figure 38: Configure individual CSP alpha values – Tutorial 1..75

Figure 39: Configure concentration values for Residue Evolution Plot – Tutorial 1....76
Figure 40: Configure Residue Evolution Plot – Tutorial 1....76
Figure 41: Setting up the Experimental Tree – Tutorial 2....80
Figure 42: Loading FASTA files – Tutorial 2....80
Figure 43: Populating the Experimental Series Tree – Tutorial 3....84
Figure 44: Settings configuration for Tutorial 3....84
Figure 45: Loading .fasta files for Y axis – Tutorial 3....85
Figure 46: Loading .pre files for Y axis – Tutorial 3....85
Figure 47: Compacted Bar plot for A27C (278K) along the Z axis. The datapoint corresponding to the paramagnetic data represents the input theoretical PRE profile. While the diamagnetic is a division over the self yielding 1....85

I. Introduction and README

a) What is Farseer-NMR?

The daily problem

Biomolecular NMR-related projects require thorough investigation of a system under study, which usually translates into testing it against **multiple experimental variables** (e.g. ligand concentration, ligand nature, temperature, pH, paramagnetic agents). Such experimental setups ultimately generate large and complex datasets of peaklists that easily overload human capacity of analysis by standard means. Treating peaklist datasets of this nature in a fast and straightforward manner is a **growing requirement for researchers**. NMR researchers benefit from many computational tools, available at the different stages of the NMR projects pipeline, which boost their capacity to extract the most out of NMR experiments in a fast and reliable way. In spite of this, one of the connections in the NMR analysis pipeline is broken due to a lack of software availability. This connection is the transformation of curated peaklists into biophysically-relevant restraints and data-rich tables and plots.

The solution

Farseer-NMR is a software package that **automatically treats, calculates and plots** NMR data and parameters derived from experiments measuring the sequential response of a system to a single or **multiple correlated variables.** The process of handling large amounts of diverse NMR data can be tedious, repetitive, error prone and time-consuming; taking days and, in some cases, even weeks. Farseer-NMR removes the tedium, minimises the effect of human error, reduces the time burden to seconds/minutes and simplifies data visualisation. Figure 1 shows the position of Farseer-NMR into the NMR analysis pipeline.

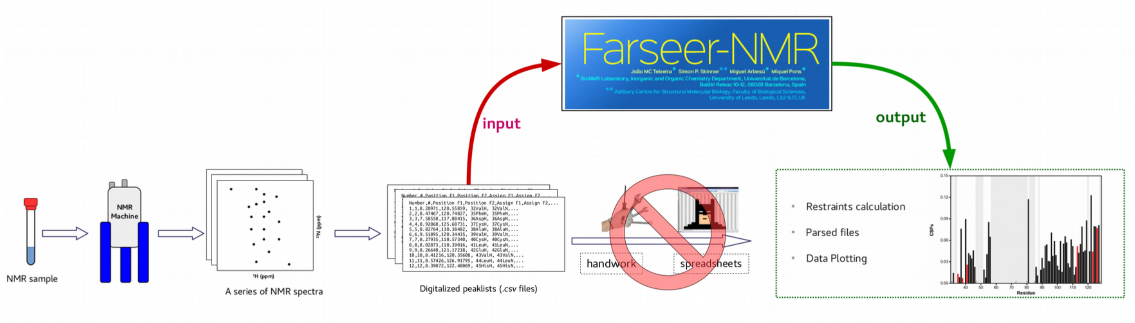

Figure 1: The BioNMR project pipeline. Farseer-NMR acts upon an old and persistent gap: the transformation of peaklists into human-readable and biological relevant data in the form of plots or parsed tables.

How?

Farseer-NMR uses high-dimensional Python 3 Numpy/Pandas arrays to **deconvolute multivariate dependent NMR data** into simpler parts, which are straightforwardly analysed and presented in a human-readable manner and without information loss.

What else?

We have implemented the most common (and some not so common) calculation routines (e.g. PRE, CSP) and several publication-quality plotting templates to improve data representation. Farseer-NMR is written completely in Python and can read the most common NMR peaklist formats: Ansig, NmrDraw, NmrView, Sparky and CcpNmr Analysis 2.4 via simple drag-and-drop import. The graphical interface is written using the most up-to-date version of PyQt, PyQt v5.8, and its modular code base enables facile extension.

b) README

Project Repository and Download

Find here the Farseer-NMR Homepage. You can download the latest release here or browse the project GitHub repository here.

Please keep in mind that this is a living and ongoing project, built by the NMR community and to the NMR community. Please expect bugs :-). We have done our best efforts to make Farseer-NMR as solid as a rock but many issues can only be identified and solved after interaction with the users and users daily problems. So, let's interact! :-)

Installation

Farseer-NMR runs on Unix based systems. Attempting to run the current version under Windows machines will fail. Farseer-NMR has been built in its entirety using Python libraries and we try to keep it up-to-date with the Python community. To install Farseer-NMR firstly:

1. Unpack the downloaded version.
2. In your Terminal emulator, navigate to the unpacked Farseer-NMR folder.

There are three different ways to install Farseer-NMR according to your system setup, here are organised from the simplest to the hardest:

1) Setting up a Miniconda for Farseer-NMR

The easiest way to setup Farseer-NMR is to install a dedicated Miniconda Python distribution inside the Farseer-NMR main folder. **We recommend this for the majority of users.** This setup demands extra disk space (approximately 3GBs) but ensures that you can run Farseer-NMR independently from your Python installation setup. Do this with the following steps:

1. Give execution permissions to Linux_install_Miniconda.sh

   `chmod u+rwx Linux_install_Miniconda.sh`

2. Run `Linux_install_Miniconda.sh`. This will install a Miniconda distribution with all libraries required to run Farseer-NMR.

   `./Linux_install_Miniconda.sh`

2) Creating a dedicated Anaconda environment

If you use Anaconda as your Python distribution you can try to run Farseer-NMR directly, most likely all the required libraries are already installed. Alternatively, if you do not want to change your main Anaconda Python environment, you can create a secondary Anaconda environment dedicated to Farseer-NMR:

1. Give execution permissions to Linux_install_env.sh

   `chmod u+x Linux_install_env.sh`

2. Run `Linux_install_env.sh`. This will create an Anaconda environment with all the required libraries to run Farseer-NMR inside the `envs/` directory of your Anaconda installation.

   `./Linux_install_env.sh`

3) Installation for advanced users

If you are an advanced user who is proficient in manually managing your own installed Python libraries, you can get a list of the libraries required to run Farseer-NMR in the architecture respective `spec-files` inside the Documentation folder. Afterwards, to create the `run_farseer.sh` file execute:

```
./Linux_install_manual.sh
```

Running Farseer-NMR

Before running Farseer-NMR, please read carefully the Installation section. After Farseer-NMR has been correctly installed, to run execute:

```
./run_farseer.sh
```

Running without the GUI

A Farseer-NMR calculation run can be launched without the GUI interface. Nevertheless we advocate the use of the GUI version for all users, advanced and beginners. All implemented and functional features are available through the GUI. We advise the use of the command line version only for developers. For further information, see here.

Warnings, Errors and Troubleshooting (WET) list

We have implemented several fail safe checks during the Farseer-NMR calculation runs to warn the users when the input data or configuration would lead to erroneous behaviour. When an error is found, Farseer-NMR outputs a short explanatory message and the user is redirected to a full explanation under the Wiki WET page.

Participate in the Farseer-NMR community

There are several ways that you can help us improve Farseer-NMR and be part of its community! :-)

Reporting a bug

If you find bugs, mis-functional or non-functional features during your calculation runs, please report them by using the Issues tab under the GitHub repository. We have prepared a set of labels that help you to specify the nature of the issues.

Suggesting Features

You can also use the Issues tab under the GitHub repository to suggest new features that you would like us to implement or that you would like help implementing.

Become a collaborator

Help us implement new features! Do you have your own NMR analysis routines that you would like to see implemented in Farseer-NMR? You can fork the Farseer-NMR project to your own GitHub account, write the functionalities and prepare a pull request for us to review and help you out! We have prepared a Farseer coding style guide for developers so that we keep code readability consistent. We kindly request that contributors to the project adhere to this style guide. Even if you're not an experienced programmer, please get contact with us and we will be happy join forces with you to introduce your functionalities!

Mailing list

Post on our mailing list for questions, discussion and help!

Social Network

Find us on:

- Twitter @Farseer-NMR
- Research Gate

License

The entire Farseer-NMR code base comes with no liability and is licensed under the GPL-3.0.

II. The Farseer-NMR data structure

Farseer-NMR performs complex analysis on series of NMR peaklists that inspect a system's dependency on (up to) three different variables (e.g. ligand concentration, ligand nature, temperature, pH, paramagnetic agents, etc…).

Example 1:

Given a protein system **P**, the binding profile of the ligand **L1** was measured at five concentrations (**C**). The same protein **P** was screened against four related ligands (**L1**, **L2**, **L3**, **L4**) and each experiment was repeated at three different temperatures (**T1**, **T2**, **T3**).

The above experimental setup embodies a set of 12 experimental series, which result from the combination of two **continuous** variables (temperature and concentration) and one **discontinuous** variable (ligand nature). In this case, a total of 60 2D-NMR experiments would be performed (5×4×3). NMR data are sensitive to the contribution of each experimental variable. In order to fully understand the contribution of each variable to the system under study, it is essential to have a flexible, yet simple way to access the data, which preserves all information content and allows the deconvolution of these complex contributions into simpler parts.

a) The Farseer-NMR Cube

To freely navigate and explore experimental datasets spanning multiple conditions, Farseer-NMR loads the whole input data to a single digital object, a Python Numpy/Pandas **five-dimensional array**; which, for the sake of simplicity, can be visualized as a cube made of 2D data points, where the three-dimensional axes of the cube (**x**, **y**, **z**) are the experimental variables (in Example 1, *ligand concentration*, *ligand nature* and *temperature range*), and each data point is a 2D-NMR peaklist (loaded as pandas.DataFrame) with the respective rows referring to the residues and columns to the experimental observables previously extracted from the user preferred NMR analysis suite. We have named this object the **Farseer-NMR Cube** (Figure 2).

Cube dimensions can have any number of data points and, they can be accessed and combined freely to generate a panoply of series, which encode the answers to different experimental questions.

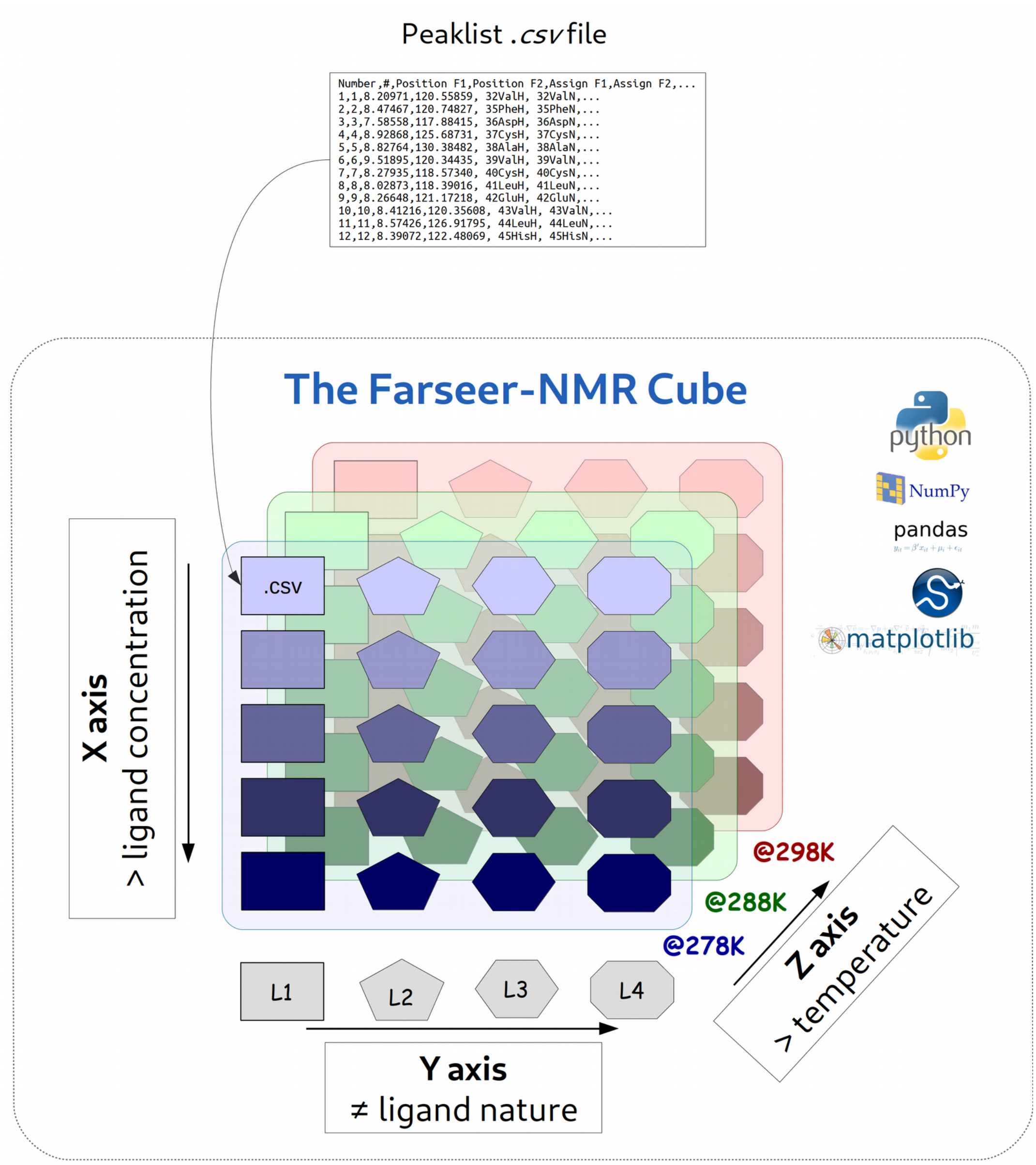

Figure 2: The schematic representation of the Farseer-NMR Cube.

b) Multidimensional Analysis Workflow

The great advantage of having the whole experimental dataset in a single digital object, the Farseer-NMR Cube, is that it can be arbitrarily sliced to investigate specific questions, which are not limited to the acquisition schedule of the multivariable data. Following the previous example, we can ask different questions of the dataset that directly relate to the conditions assayed:

1. *Ligand concentration range:*
    a. Where does the *ligand* bind to the *protein system* **P**?
    b. Are there multiple binding sites or allosteric effects?
    c. What are the *ligand concentration* dependencies of these effects?
    d. What is the binding constant?
2. *Ligand Nature:*
    a. What is the binding profile of the various *ligands*?
    b. Do the ligands interact with the same binding site?
    c. Do they evoke the same changes in **P**?
3. *Temperature variations:*
    a. How does the *temperature* affect the binding profiles of the ligand library?

As explained above, the Farseer-NMR Cube's three dimensional axes correspond to the evolution of the system as a function of the three experimental assayed conditions and, therefore, we can explore the above cited questions by slicing the cube along the different axes, where the above points **1**, **2** and **3** correspond to Farseer-NMR Cube's axes **X**, **Y** and **Z**, respectively.

A Series of NMR experiments

A series of NMR experiments is any set of experiments (peaklists) that represent the evolution/change of a system or experimental response as a function of an external variable, which can be *continuous* or *discontinuous*, where the first experiment is the reference against which all the other experiments are consecutively evaluated.

Following the above rationale, and considering a multivariable dataset of peaklists represented in the Farseer-NMR Cube, we can fix two points along two given axes (e.g. X=**C2** and Z=**T2**) and slice along the third axis (**Y**) to generate an 1D-vector of 2D-NMR peaklists, which would correspond to the experimental series **[Z=T2][X=C2][Y=[L1-L4]]**; where Y=L1 is the reference experiment of the series.

From the Farseer-NMR Cube, we can extract as much sets of 1D-vectors (series of peaklists) as different combinations of the three axes X, Y and Z.

Following example 1, out of the 12 experimentally-acquired series relating to the progression of ligand concentration (4×3, in our example), Farseer-NMR can extract up to 47 *in silico* generated series of experiments that result from the different combinations of the other experimental variables:

- 4×3 (ligand concentration)
- 3×5 (ligand nature)
- 5×4 (temperature dependence)

Navigating the Farseer-NMR Cube's Axes

The implemented workflow sequentially generates *series of experiments* (peaklists) out of the Farseer-NMR Cube, which result from all possible combinations of X, Y and Z. This is accomplished by fixing data points along two axes and extracting the series along the third one. In other words, say, the progression of X as a function of a given Y and a given Z.

Evolution along the X axis

Farseer-NMR analyses how the whole dataset evolves along the X axis by fixing one data point in Y and another in Z and creating a series of peaklists along the X axis. A full set of series is generated by walking through the Y and Z data points (Figure 3). Following example 1, a total of 60 NMR experiments (peaklists) are organized in a Farseer-NMR cube with dimensions 5x4x3 (XxYxZ). Analysing the dataset along the X axis generates 12 experimental series that will be analysed individually according to the Analysis Routines.

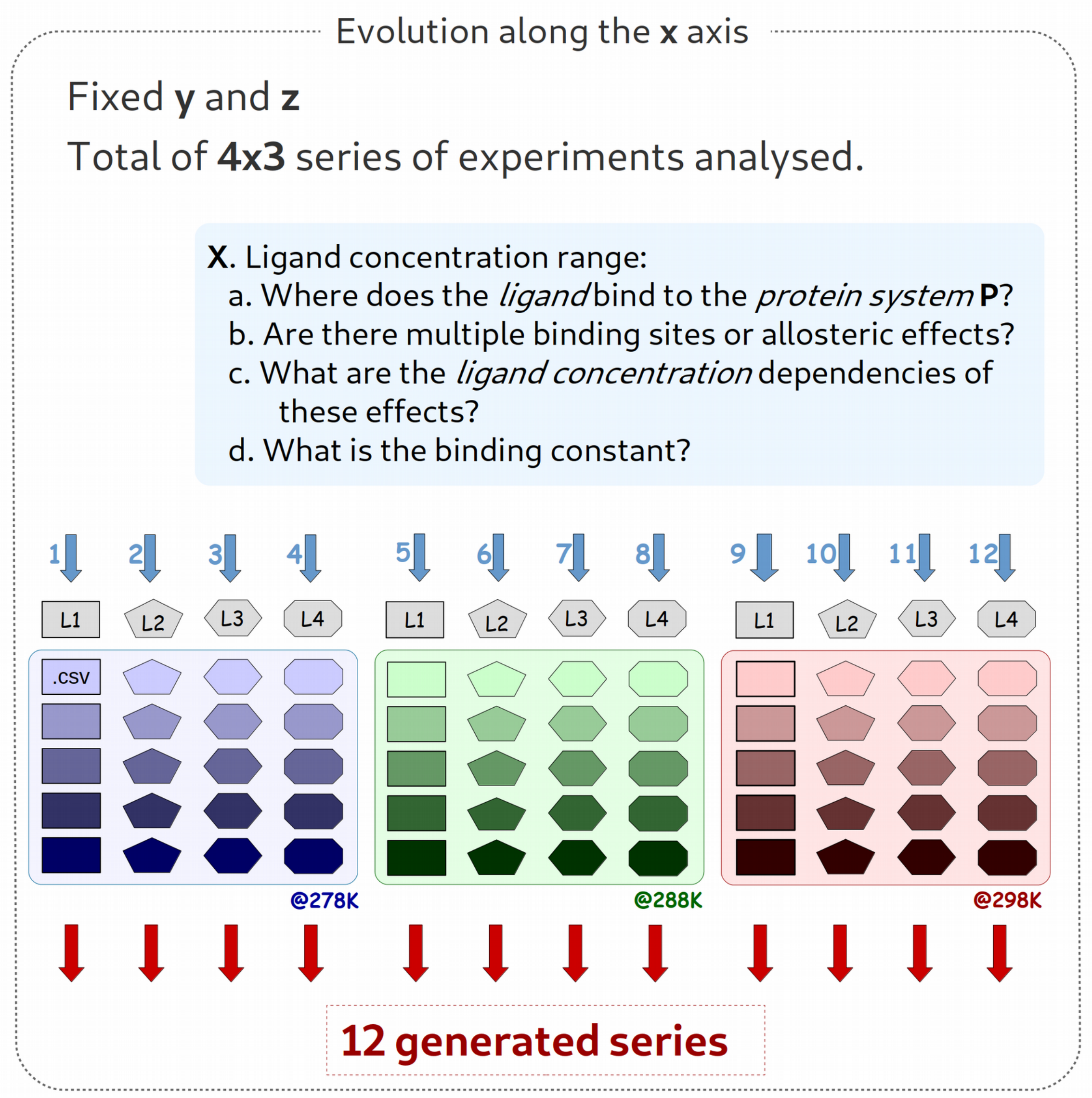

Figure 3: Representation of how the X axis series are generated along the Farseer-NMR Cube.

Evolution along the Y axis

Following the same rationale as for Evolution along the X axis, in this case, Z and X are fixed and series of peaklists are generated along the Y axis (Figure 4).

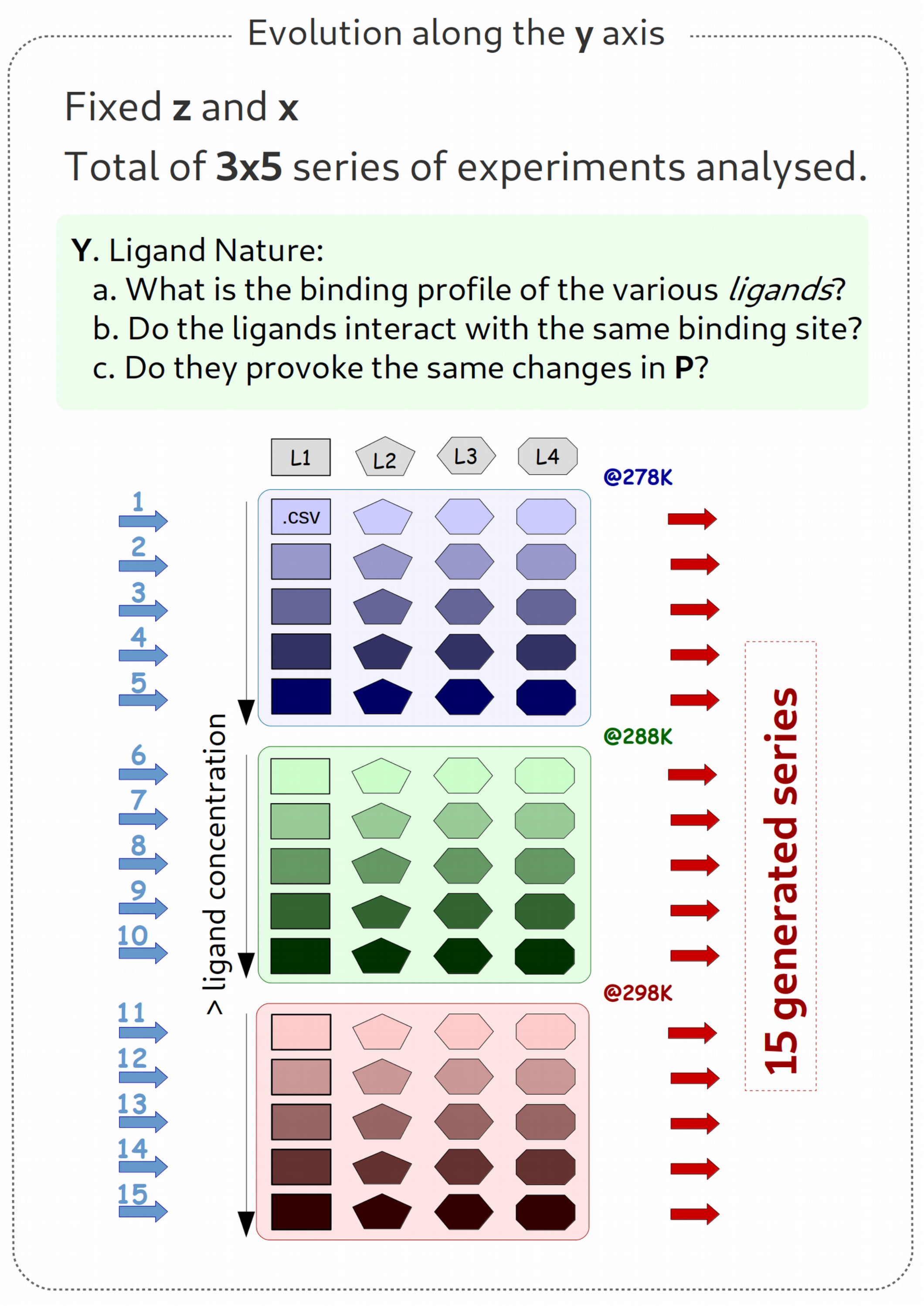

Figure 4: Representation of how the Y axis series are generated along the Farseer-NMR Cube.

Evolution along the Z axis

Following the same rationale as for Evolution along the X axis, in this case, X and Y are fixed and series of peaklists are generated along the Z axis (Figure 5).

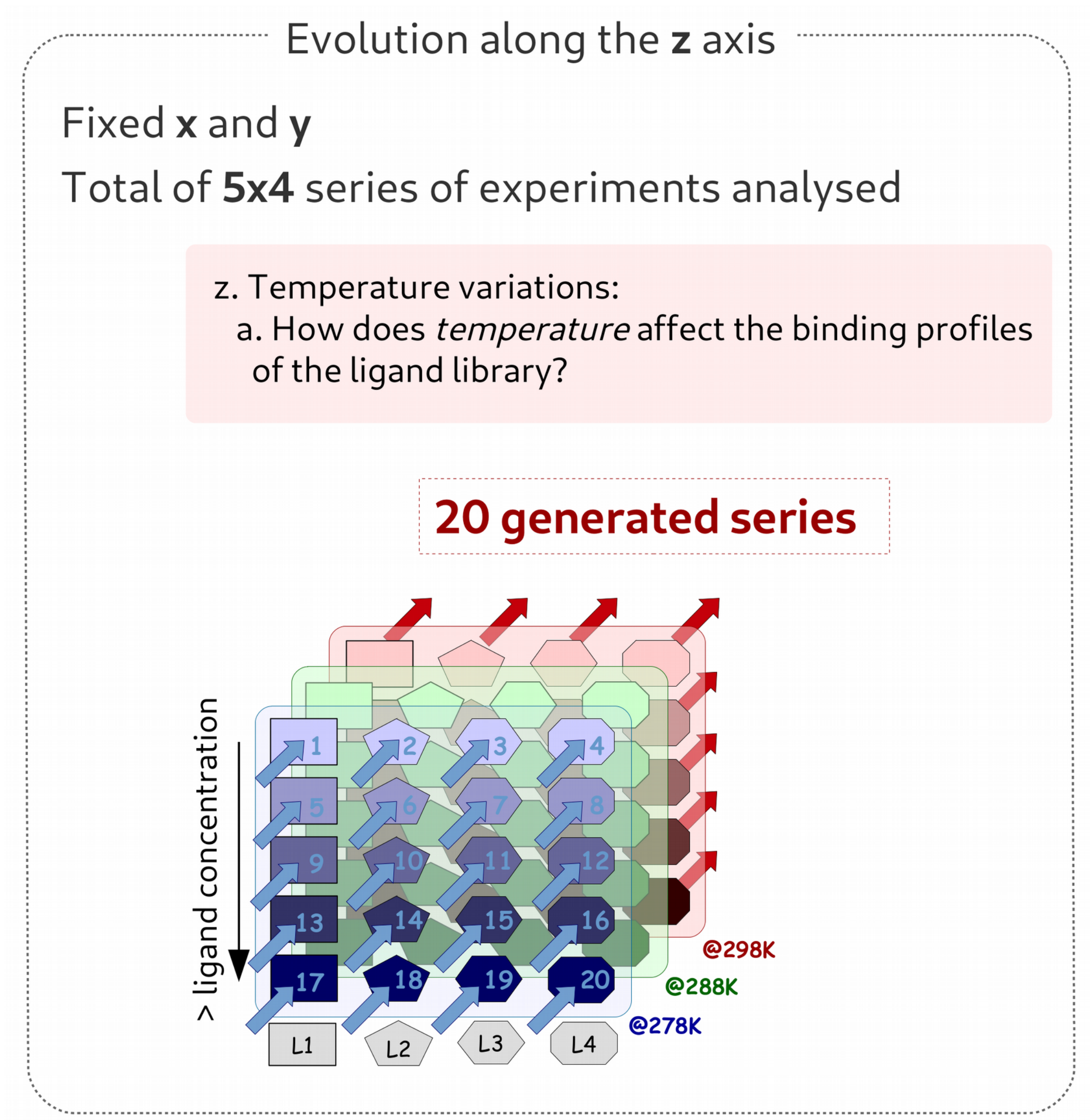

Figure 5: Representation of how the Z axis series are generated along the Farseer-NMR Cube.

Analysis of Experimental Series

Experimental series are analysed by evaluating each peaklist in the series to the series' reference peaklist. The different NMR parameters calculated (chemical shift perturbations, intensity ratios, etc.) are then stored directly in each peaklist DataFrame and conveniently exported in the different available formats (tables, plots, …) carefully structured into hierarchical folders. Farseer-NMR contains a set of analysis routines that can be applied to extract the most out of each NMR peaklist series.

c) The Farseer-NMR Analysis Routines

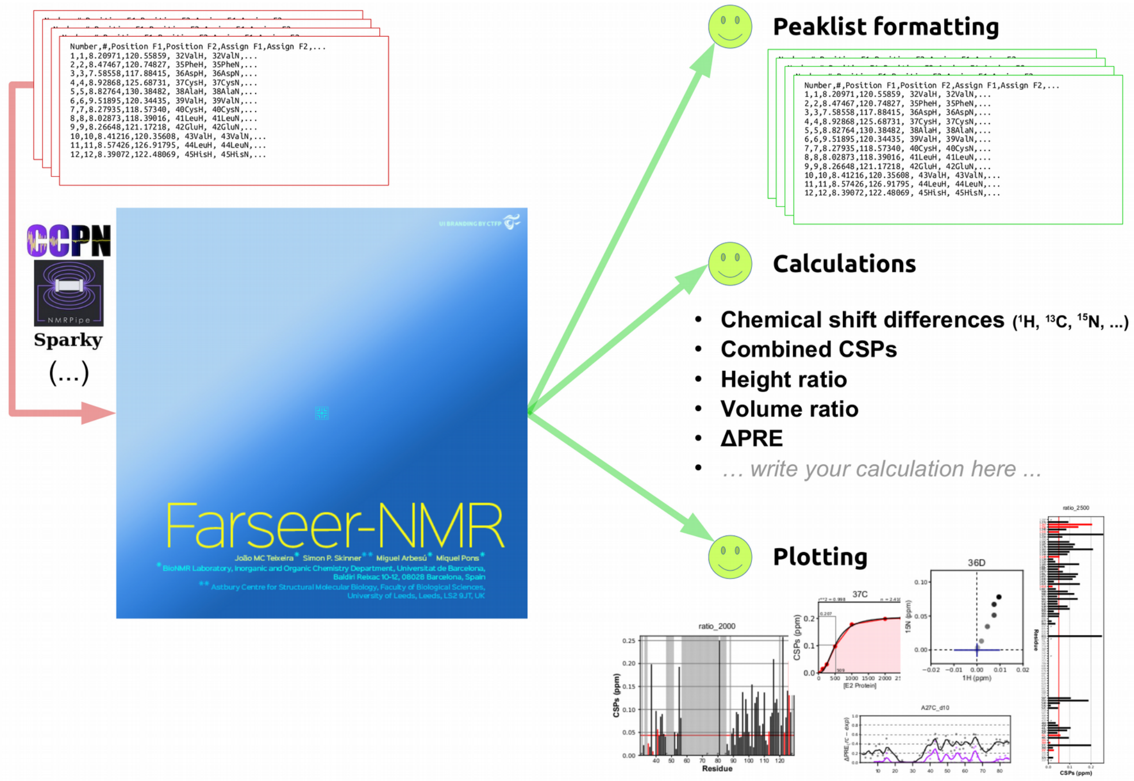

Figure 6: The schematic representation of Farseer-NMR workflow and analysis routines.

Series of NMR peaklists are extracted from the Farseer-NMR Cube following to the Multidimensional Analysis Workflow and according to the user-defined preferences. Each series can be viewed as a vector of two-dimensional NMR peaklists (data tables) that represents the evolution of a system as a function of an experimental variable, which can be continuous or discontinuous. In this vector, the first point in is the reference peaklist followed by the other peaklists in the series. Each series is analysed according to a workflow of routines, the **Farseer-NMR routines**, which can be summarised in three main steps:

1. General parsing and formatting, where all the peaklists are expanded to the same size through identification of missing and unassigned residues.
2. For each series, NMR parameters are calculated from the NMR observables, showing the evolution of these along the series.
3. Results are conveniently plotted and exported in user-friendly parsed data tables organised in dedicated folders.

Treating and Formatting peaklists

Before biologically relevant NMR Parameters can be calculated, peaklists in a Farseer-NMR Series must be treated and formatted and an initial set of information must be parsed out and organised.

The combined workflow of the routines presented bellow serve three purposes:

1. Parsing assignment information to a higher readability level
    a. Reading Assignment Information
2. Identification of missing and unassigned residues
    a. Identifying the *missing* residues
    b. Identifying the *unassigned* residues
3. Expansion of all loaded peaklists to the same size in rows and columns, this allows direct evaluation and comparison by computational means.

Technical consideration

For the sake of clarity, the above mentioned routines can be understood as being executed during at the Farseer-NMR Series level, after extracting a series from the Farseer-NMR Cube. However, these are actually performed before the creation of the Farseer-NMR Cube and while the peaklists are being organised hierarchically in the PeakList Tree, even though the end result is the same, except for very specific cases. The exact algorithm can be described as follows, practical considerations are also explained:

1. By default, identification of *missing* residues is performed only along the X axis by evaluation of each peaklist in the X axis series with the reference.
    a. To expand this analysis to the other axes, the user must activate the search for missing residues Y/Z flag. This option is especially useful when analysing paramagnetic data, because it allows tracking of peaks that disappear solely by the introduction of the paramagnetic tag.
2. Identification of *unassigned* residues based on FASTA files takes place only along the X axis, which means that FASTA files are unique for each Y data point; as described, different FASTA files can only be input along the Y axis.
    a. This can be overcome by activating the corresponding flag in search for missing residues Y/Z menu.

Reading assignment information

NMR peaklists are simply tables were rows represent residues and columns contain all the information regarding residue identification, NMR observables and notes.

In the preferred peaklist format of Farseer-NMR (CCPNMR v2 format), assignment information is concatenated into a single column (*Assign F1* in the format *1MetH*) and it is therefore necessary to split this information into a more usable format. Three additional columns are created (*Res#*, *1-letter*, *3-letter*) to store the assignment information and this information is used to index all the data and resulting calculations (Figure 7).

Assign F1
0GlyH
1MetH
2AspH

→

Res#	1-letter	3-letter
0	G	Gly
1	M	Met
2	D	Asp

Figure 7: Expansion of Assign F1 column to three new assignment informative columns.

Identifying missing peaks

When performing a series of NMR experiments as a function of an external variable, it is common to lose track of a certain number of peaks, this might due to linewidth broadening or to peak overlap. As a consequence, extracted peaklists most likely differ in size, i.e. different numbers of rows. We describe disappearing peaks as *missing* peaks. It is of great importance to identify these peaks (better put, missing peaks) as they represent the regions most sensitive to the environment, therefore, they are information rich.

Technical note: The difference in peaklists size greatly hinders direct and straightforward analysis of the peaklists files in traditional plotting tools, *because row identities won't match when comparing row by row*. In the past, this issue had to be handled manually, whereas Farseer-NMR does it automatically.

The second task of Farseer-NMR is to identify *missing* residues and, thus, expand all the peaklists in a series to the same size of the reference (number of rows). New rows are added for each *missing* residue found, and new column is created, named **Peak Status** column, to tag residues as measured or missing according to their nature. Other columns are filled with user defined default values or numpy.nan values (Figure 8).

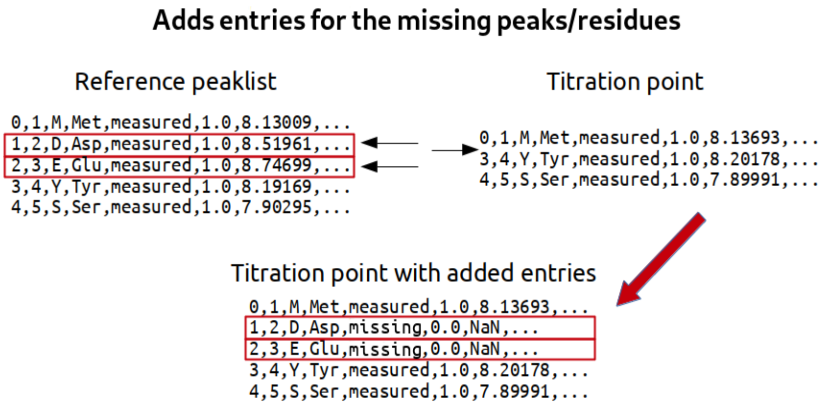

Figure 8: New rows are added to identify the peaks that were missing with respect to the reference experiment.

Identifying unassigned residues

This feature is optional and is performed in a similar manner to the identification of the missing residues, with the exception that all the peaklists in the series (reference included) are compared to a previously loaded FASTA file containing the protein's full primary structure. New rows identify the *unassigned* residues and the tag **unassigned** is added to the **Peak Status** column.

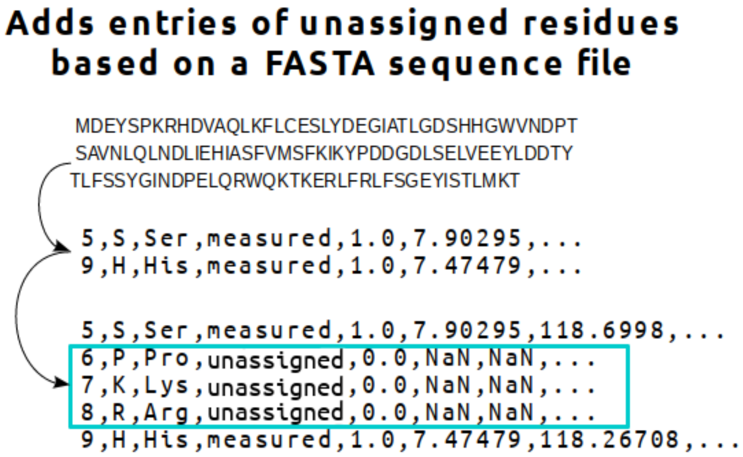

Figure 9: New rows are added to identify those residues that are not assigned in the reference, and in consequence, in the whole series.

Performing Calculations

NMR Parameter calculation, or any related routine, is performed by evaluating each data point (peaklist) in the Farseer-NMR series being analysed to its reference experiment (peaklists), which is the first in the series of peaklists.

The results are added to newly-generated columns in the peaklists' pandas.DataFrames, which are all exported together at the end of a run. Farseer-NMR can calculate:

1. Chemical shift differences for each nucleus
2. Combined Chemical Shift Perturbations (CSP)
3. Intensity ratios
4. Data Fitting
5. ΔPRE

Plotting the results

Farseer-NMR contains several publication-quality plotting templates to represent calculated data. For each series analysed, the calculated NMR parameters can be plotted in any and all available templates. Each template is highly customisable and enables a user to adapt the representation of data to different publications' requirements.

d) Comparative/stacking Analysis

Comparative Analysis, also described as stacking analysis, does not generate new data. Rather, it consists in parsing algorithms that reorganise and stack the Farseer-NMR results differently. *Note: comparative analysis generates additional pseudo dimensions in the [Farseer-NMR Cube,](#) which are essential to analyse, for example, [paramagnetically-derived restraints](#).*

Example 2:

*A protein **P** was investigated against five progressive concentrations of two similar, yet not identical, ligands (**L1** and **L2**), at 298K. The Farseer-NMR Cube of this data set has the following form (Figure 10):*

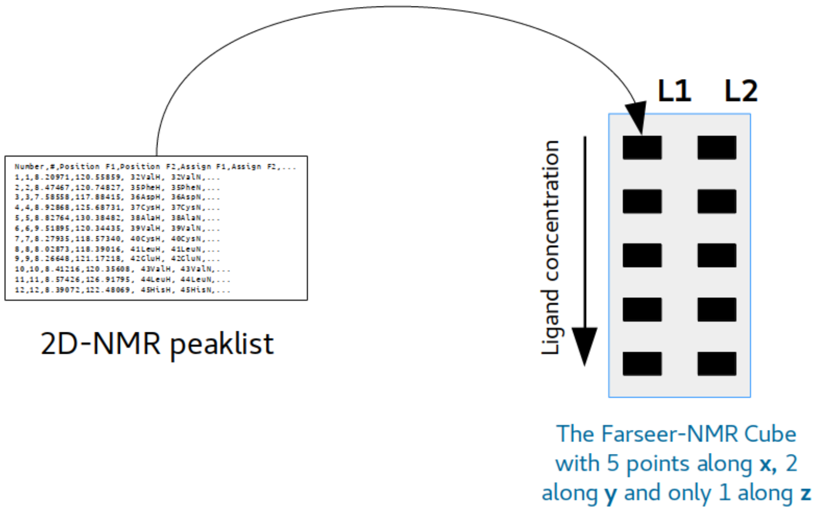

Figure 10: An example of two experimental series that represent the evolution of P as a function of the concentration (X axis) of L1 and L2 (Y axes).

*The **z** dimension consists of a single datapoint (298K), and therefore can be disregarded (although, technically, it exists). Consider the case where the user wants to analyse the two experimental series for **L1** and **L2** only along the **x** axis of the Cube, that is, in terms of increasing ligand concentration. In this case, two folders containing the corresponding results for **L1** and **L2** are generated ([further reading on folder organization](#)) containing all the tables and plots requested by the user ([analysis routines](#)).*

The generated plots, within each folder, will represent the evolution of the experimental series in response to increasing *ligand concentration*. However, it can be fruitful to compare the results obtained for the two ligands at a given concentration, in other words, have that information gathered in a single figure/file/table.

This requirement could be fulfilled by the user by simply opening the two plotting figures (or hard copies) and placing them side by side for comparison. This manual procedure would quickly become awkward when comparing large experimental datasets in which several points were acquired for a particular variable: e.g. screening a library of 20 ligands. To solve this issue, Farseer-NMR has implemented a special routine.

Following on [from the above example](#), the data generated along the X axis can be compared/stacked along the Y axis, because there are two datapoints along the latter dimension, but it cannot be compared/ stacked along the Z axis (temperature), because there is only one point on this axis and *there is nothing to compare with*. The subsections below describe the different possibilities that can be explored in Comparative/Stacking analysis.

Calculating along X and stacking along Y

Farseer-NMR **comparative analysis** generates **stacked** plots and tables along the Y axis for **each** of the datapoints of the X axis, containing the results **previously** obtained from the evaluation of the extracted series along the X axis (ligand concentration), as shown in Figure 11. **N.B.:** Comparing along the Y axis is different from analysing an experimental series along the Y axis! In the former no new data is created while in the latter new NMR parameters are indeed calculated.

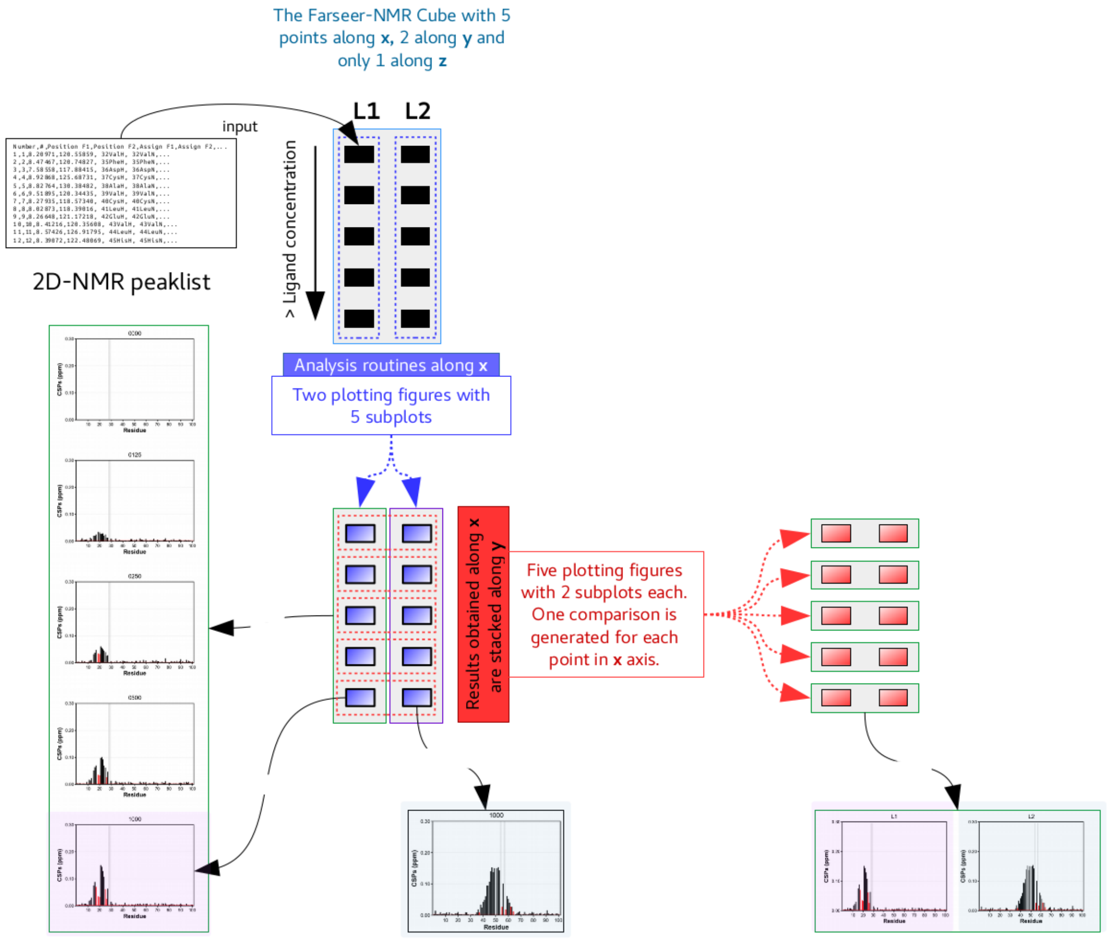

Figure 11: Schematic representation of how results generated along the X axis are compared along the Y axis.

Calculating along Y and stacking along X

Similar to the above, NMR parameters data, tables and subplots resulting from calculations along the Y axis are stacked along X.

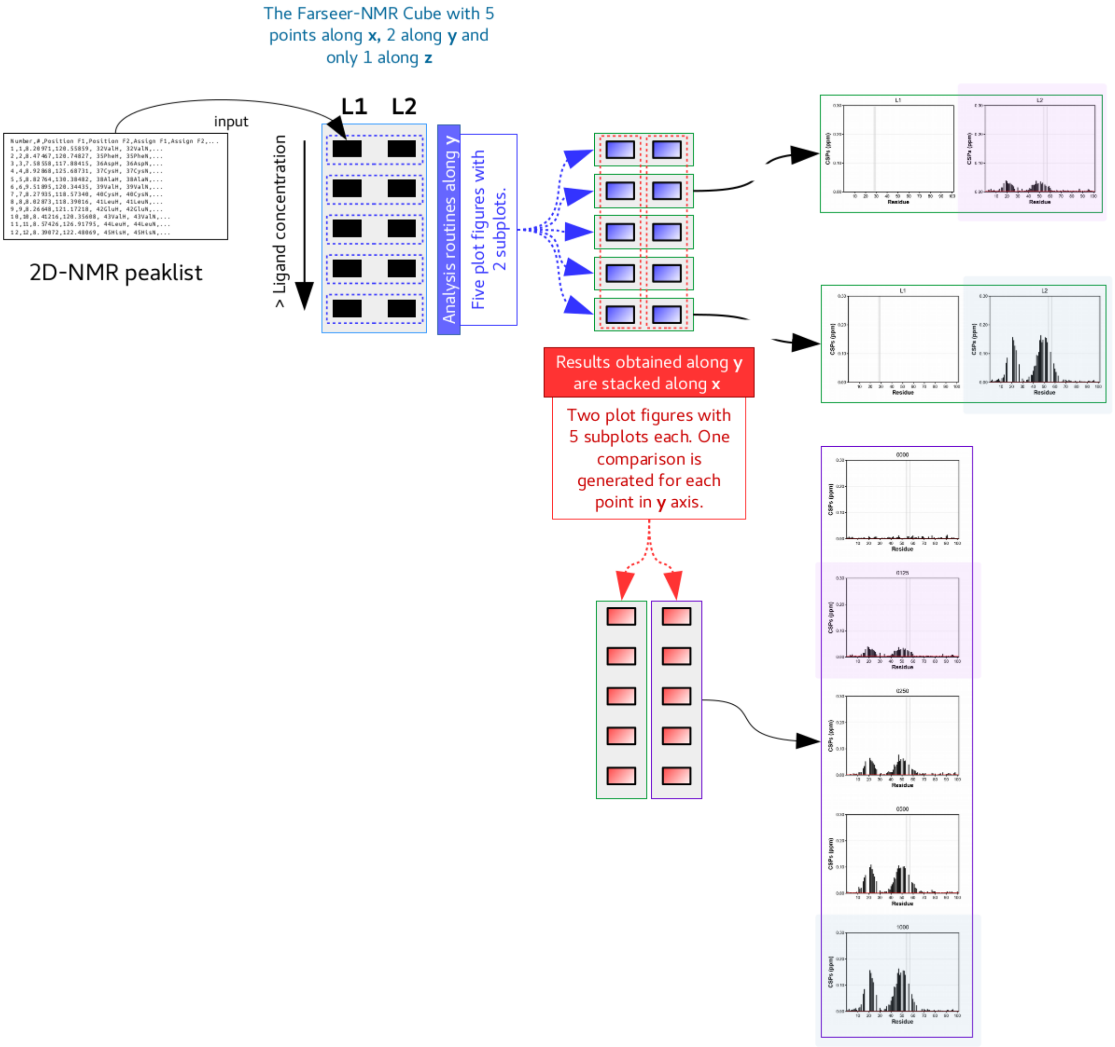

Figure 12: Schematic representation of how results generated along the Y axis are compared along the X axis.

Calculating along Z and stacking along X

Similar to the above described, subplots resulting from calculations along the Z axis are stacked along X.

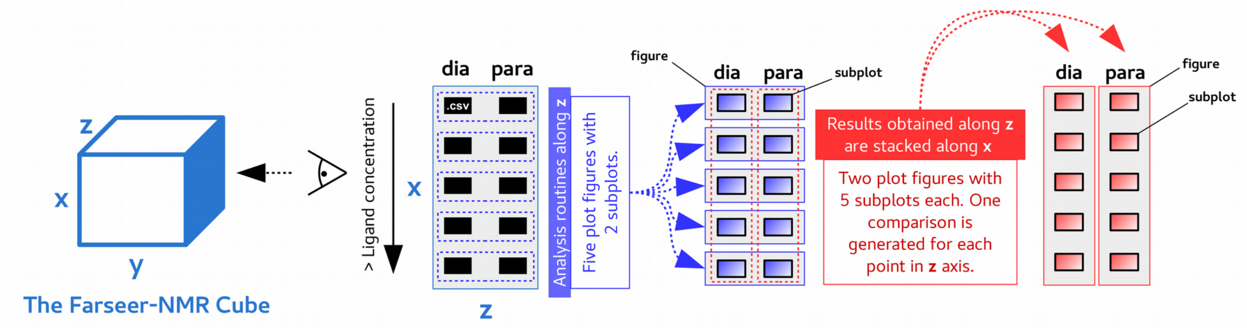

Figure 13: Schematic representation of how results generated along the Z axis are compared along the X axis.

Stacking for each dimension/experimental variable.

The same rationale can be applied to any dimension. Therefore, for each condition axis, stacking can be made along the other two axes, as long as the axis along which we want to perform the stack has more than one datapoint. Consider the previous example where 12 experimental series summing 60 HSQC experiments investigating the system's dependence on 3 different variables – Farseer-NMR Cube has dimensions of 5×4×3.

We have seen that in total 4×3 series can be analysed **along the x axis**. Stacking these results along the Y axis will generate 5×3 newly parsed series (not new data) and, stacking performed along the Z axis results in 5×4 additional series of parsed data.

III. The Farseer-NMR Program

The Farseer-NMR program consists of two main blocks: the **Core** and the **Graphical User Interface (GUI)**. The Core contains all the libraries, functions and algorithms for data treatment, organization and output generation. While the curated GUI organizes all the available functionalities in a human-readable and convenient graphical menus that allow users from all expertises to take the maximum benefit from Farseer-NMR.

The two coding blocks function independently and communicate with which other only via a configuration file in JSON format. The GUI can load and save the settings to the `config.json` file that serves as input to the Core when a calculation is launched.

In this section you will find general information on how to setup a Farseer-NMR Run via the Graphical User Interface, our main and recommended way of using of Farseer-NMR. It is possible also to run Farseer-NMR via command line using only the Core code, though we advise this use only for developers and, even then, only in certain cases.

Further sections are dedicated to explaining the implemented calculations, additional functionalities, organization of the generated output and the plotting templates available.

Section VII-Tutorials provides real case examples with extended explanations on how to use Farseer-NMR.

a) The initial input structure

Farseer-NMR performs complex analysis on NMR peaklist datasets by generating experimental series (of peaklists) from permutation of the experimental variables defined as described in the Multidimensional Analysis Workflow subsection.

To initiate a Farseer-NMR run, and hence create the Farseer-NMR Cube, data should be input in the form of a hierarchical tree that comprises the three experimental variables (Cube's axes): X, Y and Z. A logical tree of experiments is created where the top level corresponds to the Z axis, followed by the Y axis data points and finally the X axis data points, see next subsection.

Example 3:

Two protein mutants (mut1 and mut2), in samples of a 50μM concentration, were measured (NMR ^{1}H-^{15}N HSQC) against ligandA in three increasing concentrations 50μM, 100μM and 200μM. Mut1 and Mut2 were labeled with the paramagnetic tag, MTSL and, therefore, two different series of experiments were acquired: an initial paramagnetic series and a diamagnetic series that serves as reference to the first one. A total of 2x2x3=12 NMR experiments were measured. After analysing the spectra and exporting the resulting peaklist files, containing all the NMR observables and residue information, we face the task of extracting biologically relevant information out of those data.

The structure of these experiments can be organized hierarchically in the following way:

```
Diamagnetic
    mut1
        LigandA 50μM
        LigandA 100μM
        LigandA 200μM
    mut2
        LigandA 50μM
        LigandA 100μM
        LigandA 200μM

Paramagnetic
    mut1
        LigandA 50μM
        LigandA 100μM
        LigandA 200μM
    mut2
        LigandA 50μM
        LigandA 100μM
        LigandA 200μM
```

This structure is the base of Farseer-NMR data structure (peaklist dataset) and is from this that the Cube will be created.

b) Setting up the Experimental Tree

To configure the Farseer-NMR Cube a hierarchical tree (Z → Y → X) must be defined and populated with the corresponding peaklists, as explained in the previous section (IIIa). Peaklists can be input in two different ways: 1) Through selection of a directory path via the **Peaklist Dataset Folder** field in **Settings tab**, which selects the folder containing the peaklists (Figure 14) or 2) they can be directly dropped into the Side bar of the Peaklists tab (Figure 15), this method can be perform in addition to method 1).

Practical considerations:

- Peaklist files in the Side Bar cannot share the same name.
- A description of the compatible peaklist file types and formats can be found here.

The number of data points in each axis can be defined as well as the name for each data point. The **Setup Experimental Series** button draws the experimental tree which must be populated with the peaklists by drag and drop from the side bar. Peaklists in the three can be placed back into the side bar back via a right mouse menu.

A maximum of 15-10-10 data points per axis X-Y-Z can be defined in the GUI. If your project requires additional data points please contact us! :-)

Logical order of the experiments

At some point during the input procedure, the user needs to tell Farseer-NMR the logical order of the experiments, that is, which is the reference and which experiments follow and by which order. The logical progression of the experiments if defined by the order input during the creation of the **Experimental Tree**. In other words, the order in which the datapoints are created defines the logical order of the experiments inside the Farseer-NMR Cube, where the first experiment (peaklist) is always taken as the reference. Further information on how this order is maintained can be found in the results folder hierarchy section.

Known issues:

- An issue related to the PyQt library is known to interfere at this stage by rendering invisible or repeating the peaklist names in the **Side Bar** and the **Tree**. If such happens simply click the **Setup Experimental Series** button to return things back to normal. We are making efforts to solve this.

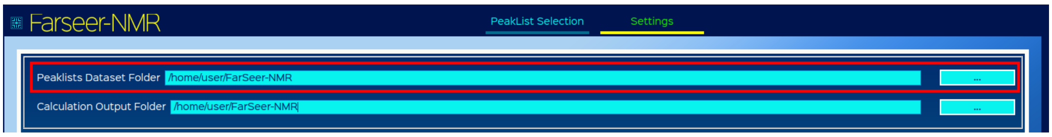

Figure 14: Cropped region of the Settings tab. Selecting the folder containing the peaklist files in the via **Peaklist Dataset Folder** button.

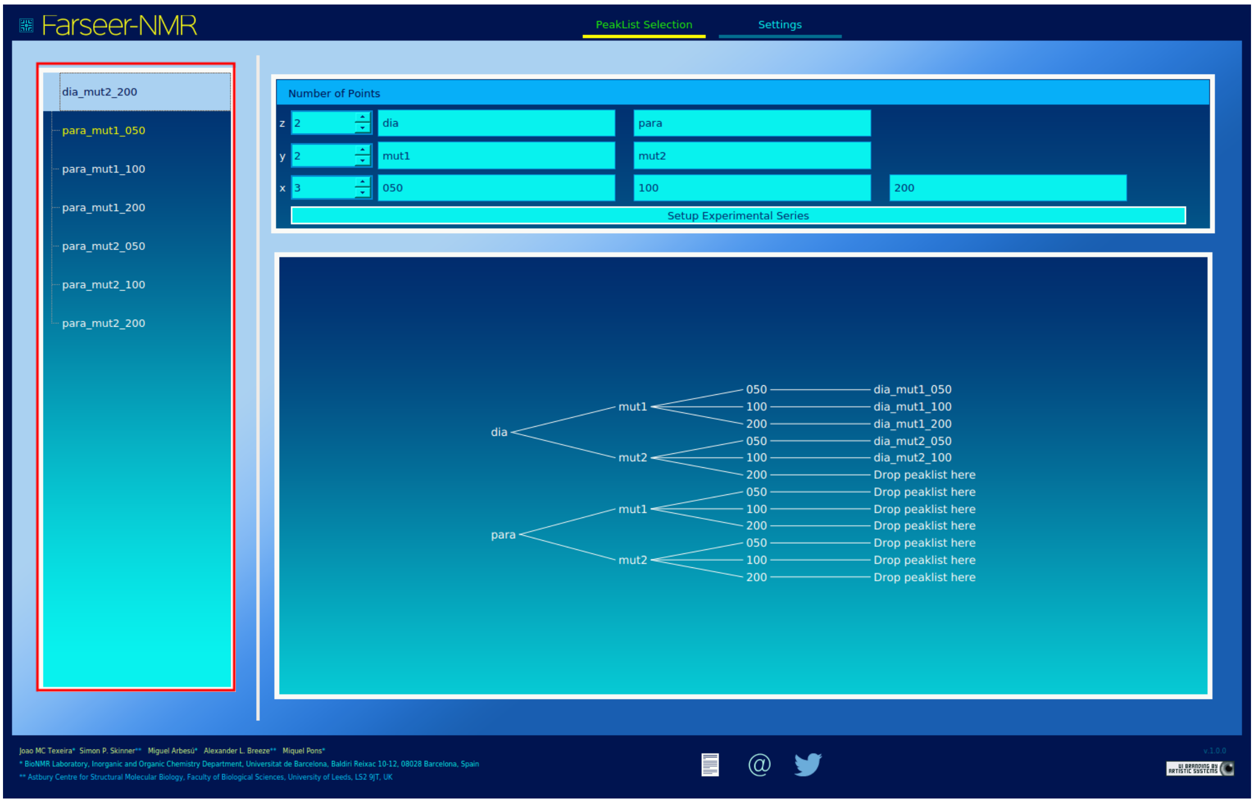

Figure 15: Adding peaklists to the Side bar, configuring the tree and populating it by drag and drop in PeakLists Selection tab.

c) Settings Tab

All the settings that have reached a stable state are available in the Farseer-NMR user interface and can be configured directly in the **Settings tab**. Experimental routines under development will be available firstly only via the JSON configuration file.

Settings are categorised in individual boxes according the routines or group of routines to which they belong. Additional, and specific, settings may be available via pop-up menus.

The **Calculation Output Folder** is the folder where the spectra will be parsed in and all the output results will be stored according to the Results Folder Hierarchy.

Plotting settings submenus are hierarchical. For example, settings under **General Series Plot Settings** apply to every plot categorised as **Series Plot**, which are the bar plots and the DPRE map.

The **Load/Save Configuration** buttons allow the user to keep copies of the configuration JSON file containing information of the whole Farseer-NMR session. A previous session can be readily recovered simply by loading a previously configures JSON file – read further.

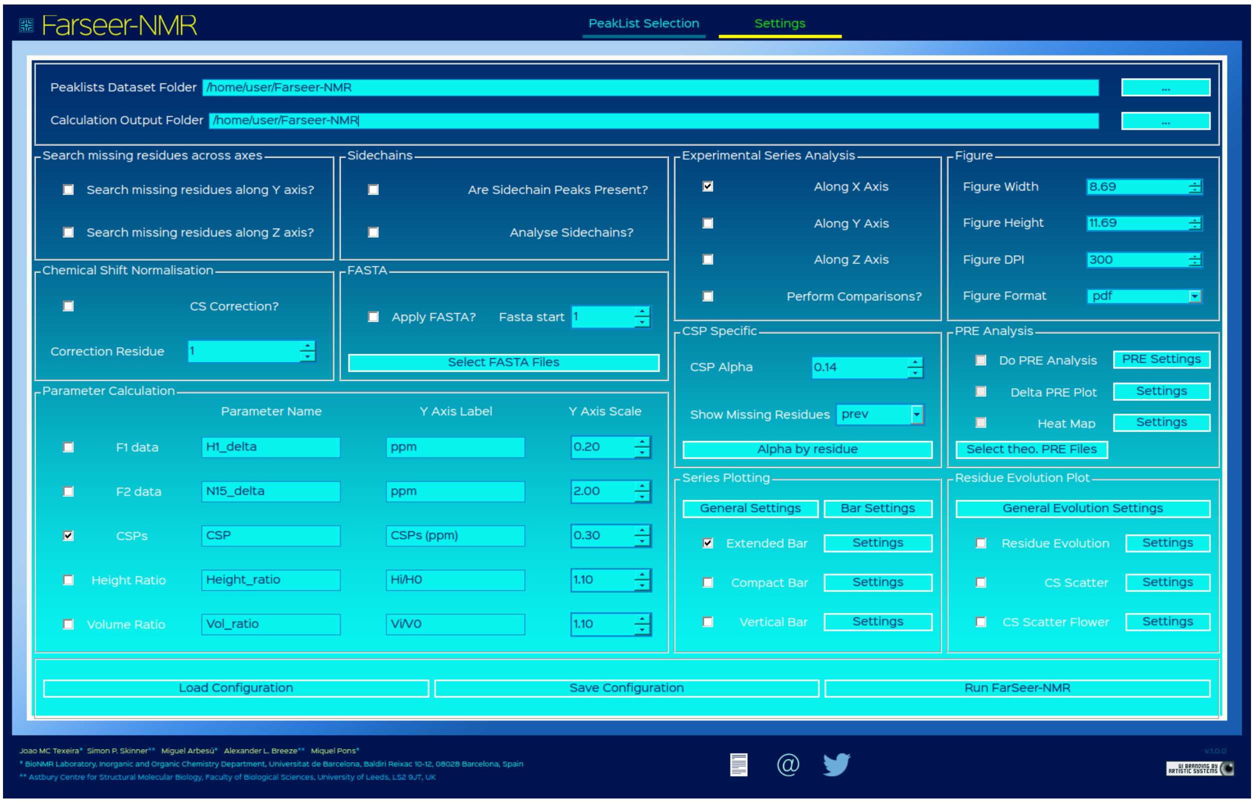

Figure 16: Farseer-NMR Settings tab

d) Running without the GUI

A Farseer-NMR calculation run can be launched without the GUI interface. Nevertheless we advocate the use of the GUI version for all users, advanced and beginners. All implemented and functional features are available through the GUI. We advise the use of the command line version only for developers.

1) Export the Farseer-NMR PYTHON PATH, where {FARSEER_ROOT} is the Farseer-NMR directory.

```
export PYTHONPATH=\PYTHONPATH:{FARSEER_ROOT}
```

To run Farseer-NMR command line:

```
python <FARSEER_ROOT>/core/farseemain.py <CALCULATION_FOLDER> <CONFIG.JSON>
```

where the CALCULATION_FOLDER is the folder containing the hierarchical spectra/ folder. Whereas the GUI will create the spectra/ folder for you automatically, to run Farseer-NMR from the Core, you have to create this folder yourself or use a previously created one, containing the peaklists *.csv files and also the FASTA files or of any other necessary files. The format of the spectra/ folder is such as is created by the GUI, explained in the core/fslibs/FarseerCube.load_experiments() method docstring or in the previous section.

The CONFIG.JSON is any configuration file saved by the GUI, with or without information describing the peaklist **Experimental Tree** as this information is not read when running command line. It is possible to change the calculation settings manually by editing the .json configuration file or loading your configuration via GUI and set it up there and saving it afterwards, there is a file with the default settings that can be used as templated, the defaulf_config.json provided in the repository under core/. Read further on the configuration file.

IV. NMR Parameters

a) Chemical shift Differences

Standard chemical shift differences are calculated separately for each atom type, currently proton and nitrogen, and for each residue. Results are stored by default in new columns named 1H_delta and 15N_delta, respectively.

Related features:

- Absolute (measured) chemical shifts can be normalised to a given residue in the reference peaklist using the Chemical shift normalisation routine.

b) Combined Chemical Shift Perturbations (CSP)

Combined Chemical shift perturbations (CSP) are calculated according to equation 8 of Williamson 2013 (and Corrigendum):

$$CSP_{(ppm)} = \sqrt{\frac{1}{2}\left[\delta_H^2 + (\alpha . \delta_N)^2\right]}$$

where δ_H is the chemical shift (cs) difference for the proton, δ_N is the chemical shift (cs) difference for the nitrogen dimension as calculated in step IV.a, and α is the normalization constant between both dimensions. By default, 0.14 for all residue types except Glycine which takes 0.2.

CSP Specific features can be configured in the corresponding submenu (Figure 17), where:

- **CSP Alpha** is the general α value
- **Show Missing Residues**, is way missing residues will be displayed in the bar plots:
    - **prev**, the last calculated value is shown
    - **full**, a full bar is shown
    - **zero**, no bar is shown
- α values can be setup individually for each residue type under the GUI popup menu **Alpha by Residue** (Figure 18).

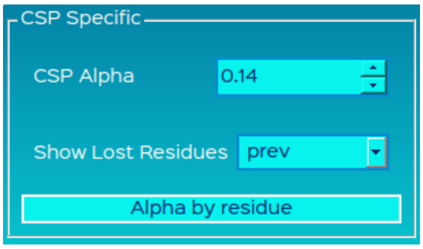

Figure 17: CSP Specific submenu.

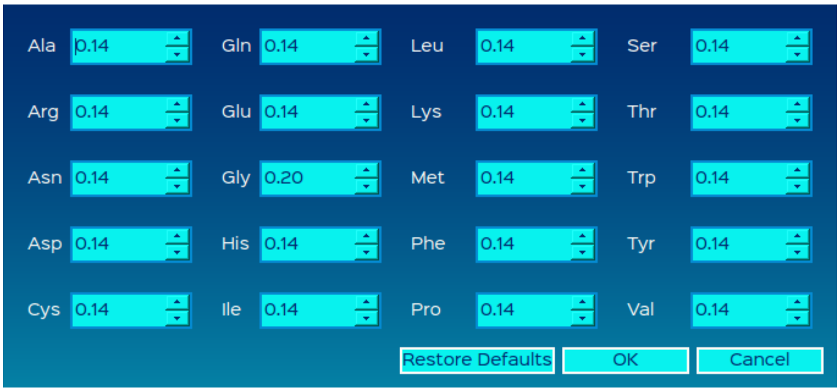

Figure 18: Alpha value by residue type popup menu. Here you can configure the exact alpha value for each residue type.

c) Intensity Ratios

Intensity ratios are calculated for the Height and Volume columns of the input peaklists, according to:

$$\frac{{}_{res}I_{p}}{{}_{res}I_{ref}}$$

where *I* is the peak intensity, either Height or Volume, *p* is the peaklist under observation, *ref* is the reference peaklist in the series and *res* is the residue under observation.

d) PRE and ΔPRE Analysis

Theoretical PRE profiles

Farseer-NMR allows the input of theoretical Paramagnetic relaxation enhancement (PRE) profiles to which intensity ratios can be compared. For that, it incorporates a set of routines that load and represent theoretical PRE profiles into the Bar Plots. Further information on how to input a theoretical PRE profiles is available in the corresponding subsection.

For the sake of organization, the practical aspects of the PRE analysis are explained in this section. To better understand the following explanation please carefully read the Results folder hierarchy section and the other sections referred.

The Theoretical PRE profiles will be displayed in the Bar Plot representations of both Height and/or Volume ratio NMR parameters. As described in the axes restrictions section, paramagnetic data can only be evaluated along the Z axis. Therefore, the theoretical PRE profiles are represented for the series analysed along the Z axis, that is, under the along_z/ folder (further information).

Or, in the case of the Comparative/Stacking analysis (see also folder description), the theoretical PRE profiles are only represented for the stacking sets Cz/ and for the 00_para/ series subset.

ΔPRE Analysis

A complete description of the ΔPRE Analysis that is implemented in Farseer-NMR can be found in Arbesú et al. 2017.

Briefly, a ΔPRE is defined by the difference between the theoretical PRE values and the PREs (intensity ratios) observed for each data point (peaklist). A Gaussian smoothed filter is applied on top of the ΔPRE data to facilitate the identification of interaction patterns.

- To activate the ΔPRE analysis, it is necessary that the calculation of **Height** or **Volume** ratios are activated.
- There are two specific plots available to represent the calculated ΔPRE values, those are the Delta PRE plot and the Delta PRE heatmap.

The output of the ΔPRE Analysis is as previously described for the Theoretical PRE Profile representation. The folder TablesAndPlots/PRE_Analysis/ will be created for the series along_z/ folder where the ΔPRE heatmap plots will be saved.

A special case is the Delta PRE map. This plot represents the evolution of the ΔPRE, which is calculated along the Z axis, as a function of another variable, represented along X or Y, this is a typical use case of Comparative/Stacking analysis. Therefore, the ΔPRE maps will be represented only under the folder Cz/ and for Z datapoint 00_para/, it appears together with the ΔPRE heatmap plots inside the TablesAndPlots/PRE_Analysis/ folder.

V. Other routines and Technical Details

This section contains information on routines available on Farseer-NMR, different from NMR parameter calculations, and summarises technical details that are important to consider to correctly set up a calculation run.

a) Compatible Peaklist Formats

Farseer-NMR can read and parse different peaklist formats, namely: sparky.peaks, ansig.peaks, nmrview.xpk and nmrdraw.peaks. You can find examples of those peaklist formats in the Documentation/Accepted_Peaklists_Formats/ folder.

For the case of NmrView/NmrDraw peaklists it is necessary to additionally provide a FASTA file and a FASTA starting number so that Farseer-NMR can link the residue number information present in the peaklists to the corresponding residue types. You can do this using the same FASTA submenu in Settings Tab. You should select one FASTA file for each Y axis condition in which you have used NmrView/NmrDraw peaklists. In case you want to use those files to investigate unassigned residues during your calculation, simply check the *Apply FASTA* box.

Refer also to WET#26 for more information.

b) Load/Save and the Configuration File JSON

The Farseer-NMR GUI allows the user to save the current state of the configuration to an external file that can be loaded, at any time, to fully restore the saved settings. Everything that can be configured in Farseer-NMR GUI is saved to the configuration file: loaded peaklists, current Sidebar state, current Experimental Tree state and all current Settings.

The configuration file is a human-readable text **JSON** file, a widely used format for data storage and transfer, especially in the world of web applications.

This file can be configured manually outside the GUI interface using any standard text editor. It can than be reloaded into the GUI where changes will be visible. Please consider that a JSON file follows a very strict syntax, and manually editing of the JSON config file may lead to errors during the run if the user is not cautious about the file syntax.

Every time Farseer-NMR launches a calculation, a copy of the current setup is stored inside the **Calculation Output Folder** under the name user_config_DATE.json, where DATE is the current data and clock time. This backup is saved regardless of whether the user has previous saved or their configuration or not. In fact, the calculation run is executed from the backup configuration file and not from the user saved file.

c) Searching for missing residues along Y and Z

In the case of Paramagnetic analysis, it is likely that upon addition of the paramagnetic tag some peaks in the vicinity of the tag will disappear. Since evaluation of missing peaks takes place along the X axis, the missing peaks in the paramagnetic peaklists (when evaluating along the Z axis) would be identified as unassigned. To avoid this, and to correctly identify these residues as missing, it is necessary to activate the search for missing residues/peaks along Z and/or Y axes, there is a dedicated flag option in the Settings tab. This procedure then performs an initial search for all the Z or Y axis series available, keeping the X axis datapoint fixed to the first experiment.

Following this, the usual procedure of search for missing residues along X will take place, for the diamagnetic and paramagnetic series separately (for example), and the residues identified initially will be taken into consideration, thus summing to the analysis along X and along each of the other (activated) axes.

d) Axes restrictions

Farseer-NMR loads all the peaklist data into a 5-dimensional array, which can be thought of as a cube made of 2-dimensional data points – the Farseer-NMR Cube. The X, Y and Z axes of the cube correspond to the different analysed experimental conditions and up to three distinct experimental conditions can be analysed simultaneously. If only one condition is analysed, the Cube is simply a vector or a set of vectors.

Farseer-NMR has been designed to enable any kind of variable to be analysed, however, some caveats apply concerning what type of analysis can be performed along these different axes.

Along the X axis – Data fitting

Farseer-NMR allows the fitting of biophysical parameters to continuous data, for instance the determination of affinity constants over a ligand concentration range. Further information about available data fitting routines can be found in its corresponding section.

Parameter fitting is only available along the X axis. It is possible to represent a concentration range series along Y and Z and perform all the analysis routines, however, the parameter fitting algorithms are not available for data represented by these axes.

Along the Y axis – Different sample constructs

Many biomolecular NMR studies involve the analysis of different protein constructs/mutants. A typical example would be:

> A series of five ligand concentrations (**L1** to **L5**) are probed against four different protein constructs/mutants (**P1**, **P2**, **P3**, **P4**), resulting in a single 5×4 face of the Farseer-NMR Cube, with ligand concentration along the **x** axis and the protein constructs along the **y** axis.

Conceptually, the two axes could be swapped and the ligand concentration could be represented along Y and the protein constructs along X. However, in order for Farseer-NMR to read FASTA files and complete the output lists with information on the *unassigned* residues, this data must be put along Y axis, since FASTA files can only be loaded for the data points of the *y* axis. Consequently, swapping the two axes would prevent the effective use of FASTA files in the analysis.

FASTA files are read only on the Y axis, but, nevertheless, Y can take any kind of variable.

Along the Z axis – Paramagnetic NMR Analysis

Paramagnetic NMR investigation of a system requires the acquisition of all data under both *diamagnetic* and a *paramagnetic* conditions. If, for example, a protein-ligand titration was performed as part of a paramagnetic NMR study, the dataset would consist of two titration series: *diamagnetic* and *paramagnetic*. The *diamagnetic* and *paramagnetic* series would fit as data points of a second axis where the *concentration range* would be fit on the first axis, e.g. X axis (see above).

Farseer-NMR contains several routines specifically for paramagnetic NMR data analysis and these routines are restricted to analysis along the Z axis. Therefore, when analysing paramagnetic NMR data, e.g. PREs, input data should be organised such that the first point in the **z** axis corresponds to the *diamagnetic* series and the second point to the *paramagnetic* series.

There is an additional restriction on the names that the Z axis data points can take and that is such the diamagnetic series should be named simply “dia” while the paramagnetic simply “para”.

e) The FASTA file

Please consider reading the section on axes restrictions for FASTA files. Farseer-NMR accepts FASTA files with extension .fasta containing the primary sequence of a protein construct and accepts the following different formats:

- optional header starting with > character
- full sequence in a single line
- sequence split over multiple lines

How to input FASTA files

FASTA files can be input via the submenu **FASTA**. The **Apply FASTA?** flag should be activated for Farseer-NMR to use the .fasta files. **FASTA Start** indicates at which residue does the given FASTA sequence starts, because that information is not present in the FASTA file itself.

From the popup menu **Select FASTA Files** you can select one FASTA file for each Y axis data point. This menu will only be available after the **Experimental Tree** has been configured (read here how to). Why one for each Y axis data point? See Y Axis restrictions for further information.

When running without the GUI

Files should be placed under each YYY folder, that is *spectra/<ZZZ>/<YYY>*, together with the .csv peaklist files.

Practical considerations

Farseer-NMR can receive different FASTA files for each Y axis data point in order to evaluate a parameter's evolution for different mutants/constructs, for example. However, in order to perform analyses along the Y axis with different FASTA files, FASTA sequences must have the same length, otherwise row numbers won't match when comparing peaklists. If no analysis is to be performed along Y, and only the analysis of several constructs is to be performed along X, there is no requirement for FASTA sequences to be the same length.

f) Chemical shift normalisation

On some occasions, chemical shifts of the whole experimental dataset need to be normalized or corrected to a given peak of the reference experiment. The **Chemical Shift Normalisation** routine normalises the absolute chemical shifts of each of the nuclei of the whole dataset to a given residue, according to:

$$ {}_{r}\delta_{p}^{N}(normalised) = {}_{r}\delta_{p}^{N} - ({}_{res}\delta_{p}^{N} - {}_{res}\delta_{ref}^{N}) $$

where *r* is the residue in consideration, *N* is the nucleus, *p* is the peaklist under observation in the peaklist series, *res* is the reference residue, and *ref* is the reference peaklist.

This normalisation routines is applied along the X axis and throughout the whole data set.

g) Theoretical PRE profiles

Theoretical PRE profiles (such as those generated by Flexible-Meccano) should be given as a .pre file where the first column contains the residues numbers and the second column contains the PRE values. The first line should be commented with a # character directly followed (without space) by the residue number where the paramagnetic tag was engineered, for example:

```
#50
1  8.469022e-01
2  8.543896e-01
3  8.474678e-01
```

The theoretical PRE profiles are selected for Y axis data points in the same manner as FASTA files from the popup menu **Select theo. PRE Files** under **PRE Analysis** submenu.

When running without the GUI

This file should have .pre extension and be placed under the folder *spectra/para/<YYY>* alongside the .csv peaklist files and the .fasta file if provided.

h) Data Fitting

In Farseer-NMR, we have designed a code platform to simplify the implementation of the user required routines for data fitting. Currently, and as a demonstration, Farseer-NMR can fit continuous data, along the X axis, to the Hill Equation:

$$Y = \frac{Y_{max}[S]^n}{K_{0.5} + [S]^n}$$

where, Y is the observable or calculated NMR parameter, S is the experimental variable (x axis), K0.5 is the half maximal constant and n the Hill coefficient.

How to setup a Fitting

For example, if an X axis series corresponds to a titration of protein with a ligand, the exact ligand concentrations at which the experiments were acquired should be input in Farseer-NMR. The input values are unit free, it is up to the user to care about the units given. The values should be input in a list format under the menu **General Evolution Settings → Titration X values**. For example, if the experimental ligand concentrations were 0µM, 25µM, 50µM and 100µM, the input should be: `0,25,50,100` if the desired units are µM, otherwise apply the given conversion.

Additionally, activate the flags **General Evolution Settings → Fit Parameter Evolution** and the **Residue Evolution Settings and activate the Use User Defined X Values?**.

Generated files

Fitting results are saved in two files that are stored together with the plots and tables for that parameter: 1) a fitting log file where all the information regarding the fitting procedure is stored (residue wise) and 2) a fitting results table (.csv file) where the summary of the fitting with the fit parameters are stored.

Fitting results are drawn in the Parameter Evolution per Residue plot template, this required that the flag to generate this plot is activated **Residue Evolution Plot → Residue Evolution**.

Implementing new functions

The method `FarseerSeries.perform_fit()` defines the fitting workflow inside Farseer-NMR, fitting routines are coded in the `fslibs/fitting.py` library. New fitting functions and routines can be implemented following the example provided in the code for the Hill fit.

i) Sidechain Analysis

Sidechain analysis is performed in the same way as regular Backbone analysis, but the results are kept in a separated folder.

Under the submenu Sidechains select:

- **Are Sidechain peaks present?** Use this option if there are sidechain entries in your peaklists and you want to parse them out and do not consider them in the calculation.
- **Analyse Sidechains?** Activate this flag if you actually want to analyse the sidechains present.

Stepwise, Farseer-NMR:

1. Identifies assignment information
2. Searches for missing residues in the datasets
3. Searches for unassigned residues (if applicable)
4. Separates the dataset into two: one for Backbone resonances and other for Sidechain resonances
5. Calculations are performed equally and in parallel for both datasets.

Results are stored in the Sidechains/ folder inside the Calculation Output Folder.

VI. Output and Data organization

a) The log file

Farseer-NMR prints the progress of the run to the Terminal window showing detailed information on all the operations performed. At the end of a run, the full log is exported to an external file written using Markdown syntax. In addition, a copy of the configuration file is stored at the end of the log file.

Use this file for tracking input errors in unexpected results. Maybe a wrong FASTA sequence? Or a misleading peaklist file name?

Moreover, when reporting errors or bugs, please provide the log file.

b) The results folder hierarchy

The Calculation Output Folder...

... is the folder that contains the spectra/ folder (which contains all the input peaklists), is defined in the GUI as the **Calculation Output Folder** under the **Settings tab**, and is where all the Farseer-NMR generated output will be stored:

1. a copy of the user defined variables
2. a log file
3. folders where results are stored

The Backbone and Sidechains Folders…

… are created in the main calculation folder and separate the results obtained for backbone atoms and those of side-chain atoms (if present). Farseer-NMR separates these two types of results because, from experience, concatenating results for backbone and side-chain atoms inside the same tables and plots results in awkward representations – both folders have the same internal hierarchy.

The Calculations folder…

… is found under the *Backbone* or *Sidechains* folders and stores the results generated for the different sets of series analysed along each Farseer-NMR Cube axis (condition).

The axes subfolders…

… contain the series originating from slicing the Farseer-NMR Cube along a specific axis, each axis representing a different condition, where *along_x, along_y* and *along_z*, refer to *X*, *Y* and *Z* axis, respectively.

Folders index prefix

The experimental and logical progression of the series, and hence of the Farseer-NMR Cube, is dictated by the order the user inputs the datapoint names during the **Experimental Tree** setup. The Farseer-NMR Core reads this logic by considering the alphabetical order of the names along the different axis. Therefore, to keep alphabetical order along the axes datapoint names, a numbered prefix is added to the datapoint folder's names, starting from `00_` which is always the reference experiment to that axes/series.

The different series

The series generated for each axis (*along_** subfolder) are hierarchically stored according to the data points fixed for each of the other two axes. Axis *parent-child permutations* follow the order:

- Z → Y → X
- X → Z → Y
- Y → X → Z

Some examples:

- The analysis generated from a series of experiments (*increasing ligand concentration*) performed at *298K* for ligand *L1*, will be stored under the following folder tree: *Backbone/Calculations/along_x/01_298K/00_L1/*.
- For the *298K* for ligand *L2*, the folder would be *Backbone/Calculations/along_x/01_298K/01_L2/*.
- Along the *Y* axis, for *ratio1* and *278K*, *Backbone/Calculations/along_y/01_ratio1/00_278K/*.

Each of these series subfolders store the results generated from the Farseer-NMR Analysis routines. Read further on how the results are stored.

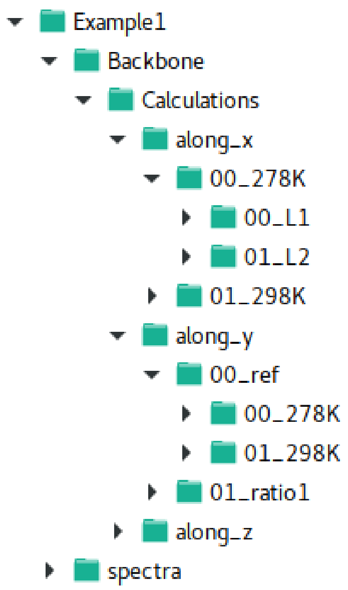

Figure 19: An example of how results for the different dimensions are organised under the Farseer-NMR data structure.

The Comparisons Folder

The Comparisons/ folder is found at the same level as the Calculations/ folder, stored under Backbone/ (or Sidechain/), and gathers the output from the Comparative/stacking Analysis method.

This folder has an additional initial subfolder, namely Cx/, Cy/ or Cz/, these indicate that the results here stored were generated by analysing the Farseer-NMR Cube along the *X*, *Y* and *Z* axes, respectively. Inside, there are along_x/, along_y/ and along_z/ folders corresponding to *the axes along which* the **comparison/stacking** was performed.

Example 4:

Calculations performed along the *X* axis and compared/stacked along the *Y* axis are stored under: *Backbone/Comparisons/Cx/along_y/.*

Recall that each comparison is by itself a series of experiments. Therefore, in this folder hierarchy you will find that the series are organized in the same manner as described above for the *Calculations/* folder. The Backbone/Comparisons/Cx/along_y/ structure is followed by a <preceding axis\>/<succeeding axis\>/<[results]> folder tree where series along these axes are stored. Below is a descriptive picture where, 00_r1/ is the first data point along the X axis and 00_278K/ is the first data point along the Z axis. 01_ratio2/ and 02_ratio3/ are other data points along X axis and 01_298K/ is the other datapoint along the Z axis. This series runs along the Y axis data points.

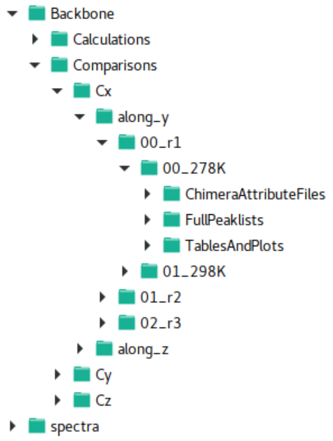

Figure 20: An example of how comparative analyses are organized under the Farseer-NMR data structure.

c) Reading the results

Inside each series folder, you will find different subfolders that organize the generated output data: tables, parsed files and plots. These are organized in different categories depending on what is stored inside. Three folders exist to organize the Farseer-NMR outputs: ChimeraAttributeFiles, FullPeaklists and TablesAndPlots.

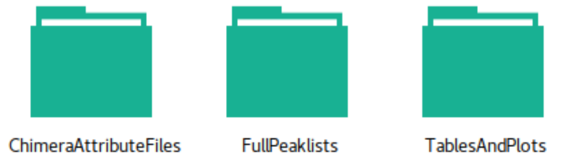

Figure 21: The folders where results from the Farseer-NMR Analysis routines are stored.

FullPeaklists folder ...

... stores the parsed peaklists in comma separated files that make up the whole series analysed. These peaklists have the same information as the originally input peaklists, plus the NMR parameters calculated during the analysis routines and, also, all the related information generated during the calculation run:

1. All peaklists are parsed to be the same length (number of rows/residues) so that they can be easily evaluated using other software.
2. Identification of unassigned and missing peaks (Proline residues included),
3. Three additional columns identifying the **residue number**, the **1-letter** amino acid code, the **3-letter** amino acid code and the **Peak Status** that identifies if the peak was measured, missing or unassigned.
4. A column for each of the calculated NMR parameters.

TablesAndPlots subfolder ...

... stores the plots created and the corresponding tables. One subfolder is created for each NMR parameter calculated (*H1_delta*, *15N_delta*, *CSP*, *Height_ratio*, ...). Inside each subfolder is a figure file for each plotting template requested and a comma separated file with the data used to generate those plots. See here for a the list of all available plotting templates.

There is also an observables folder that organizes the evolution of the NMR observables present in the original peaklists but isolated and parsed along the series. In this way, the user can make further representations of the evolution of the NMR observables simply by using the tables provided by Farseer-NMR.

ChimeraAttributeFiles folder ...

... stores Chimera Attribute parsed files that can be directly used in UCSF Chimera to represent NMR parameters into representation features (colours, ...), and contain the calculated data for each restraint and each titration data point.

d) Plotting Templates

Farseer-NMR contains a set of plotting templates that represent the calculated data in a simple, organized and **publication-ready** manner. There are plots that represent commonly used styles, and others that we have specifically designed and implemented for improved data representation.

Each generated figure represents the evolution of an NMR parameter along the whole series, either in different subplots (inside the same figure) or concatenated into a single plot.

The structure of the figures, subplot organisation in columns and rows, colours, font types and several other plotting style options are highly customisable under in the corresponding GUI menus or the JSON configuration file.

Below is a dummy example, where a randomly generated protein of 100 residues is probed against different concentrations ratios of a ligand (1:0, 1:0.125, 1:0.250, 1:0.5, 1:1, 1:2, 1:4) which cause chemical shift perturbations in a specific region.

Bar Plots

Bar plots represent the evolution of a calculated restraint in configurable and commonly used form. There are three bar plot templates available: **compacted**, **extended** and **vertical**.

General features:

- all text is customisable (font type, size and style)
- X and Y ticks and scales are customisable
- customisable colours for identification of missing, unassigned and measured bars
    - missing residues can be represented in three different manners ('full', 'prev' or 'zero')
        - 'full', represents a full bar
        - 'prev', represents the value of the previously measured point
        - 'zero', represents no value
- customisable bar width
- identification of Proline residues (boolean flag)
- user-defined labeling of bars
- user-defined colouring of bars
- a grid option
- a significance threshold line

Compacted Bar Plot

Compacted bar plots are designed to fit a half-page width figure in a scientific publication and are generally drawn in an overall figure of a columns vs rows matrix of subplots (Figures 22 and 23).

Specific features:

- summarised x axis ticks
- shadowed regions to represent *unassigned* residues.

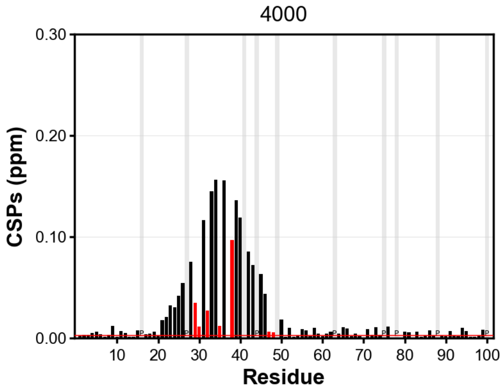

Figure 22: Compacted Bar template subplot

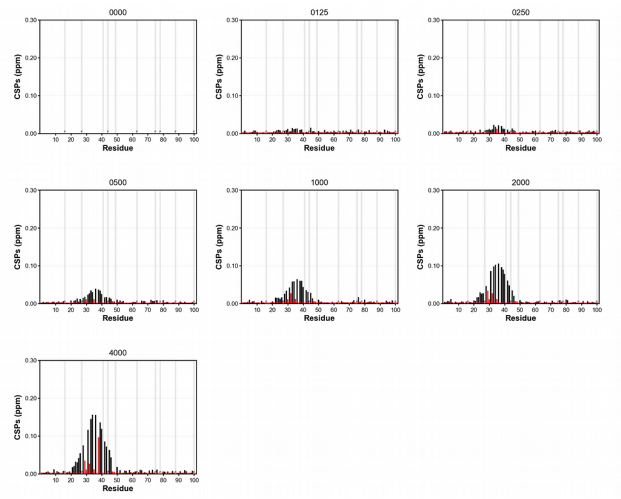

Figure 23: Full picture of a 3×3 subplot table representing the CSPs evolution of the whole series.

Extended Bar Plot

The extended bar plot is designed to fit a whole page width figure in a scientific reviewed publication and are generally drawn as overall figures of vertically stacked subplots representing the titration evolution (Figures 29 and 30).

Specific features:

- bars individually identified by residue labels up to 100 labels (larger proteins get progressively summarized ticks)
- customisable x ticks colours

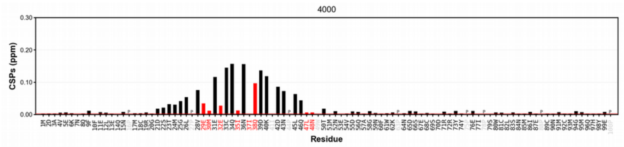

Figure 24: Extended Bar template subplot

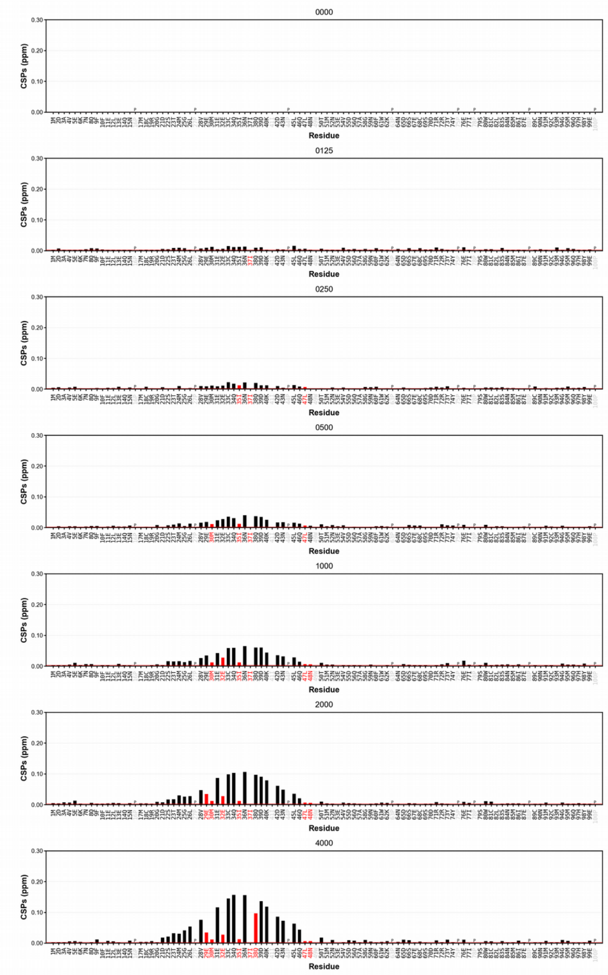

Figure 25: Full picture of a 7×1 subplot table representing the CSPs evolution along the whole series.

Vertical Bar Plot

The vertical bar plot is designed to fit narrow spaces and column organisation styles in scientific publications and are generally drawn as overall figures of horizontally stacked subplots representing the titration evolution (Figures 26 and 27).

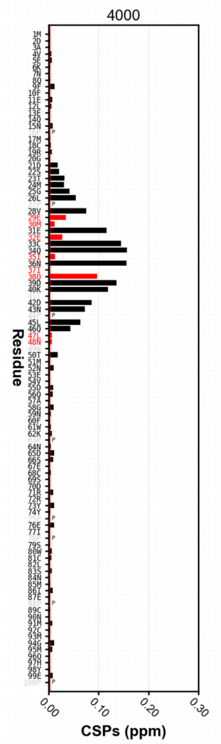

Figure 26: Vertical Bar template subplot

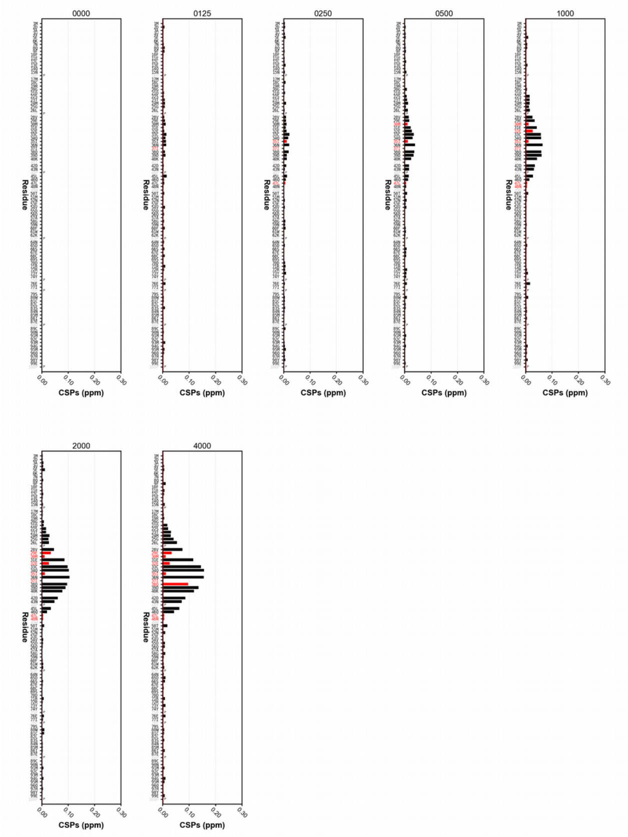

Figure 27: Full picture of a 5×2 subplot table representing the CSPs evolution along the whole series.

Residue Evolution Plots

Restraint Evolution

Residue evolution plots represent the evolution of a given restraint over the whole titration for individual residues. The generated figure is amassed into one subplot for each residue in an *MxN* matrix. It is designed to fit a page width figure in the *Supporting Information* of a scientific manuscript. Individual plots can be cropped externally and used in specific figures of the main article body. Data represented in this manner also can be fit to a given equation.

General features:

- allows data fitting!
- all text and labels are customisable (font type, size and style)
- X and Y ticks and scales are customisable
- customisable colours:
    - shades
    - plot colour
    - fit curve colour
- customisable lines width
- identification of unassigned and *missing* residues
- *missing* residues have no data point in plots

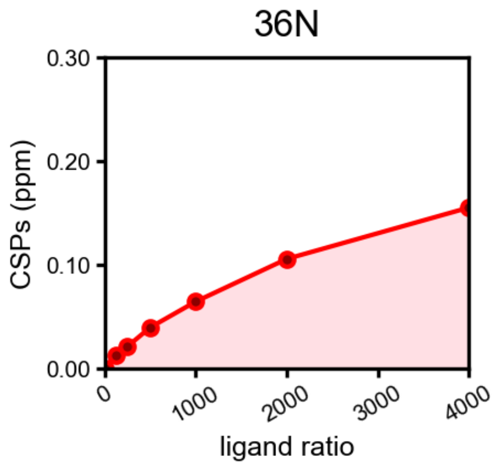

Figure 28: Subplot template of the restraint evolution representation per residue.

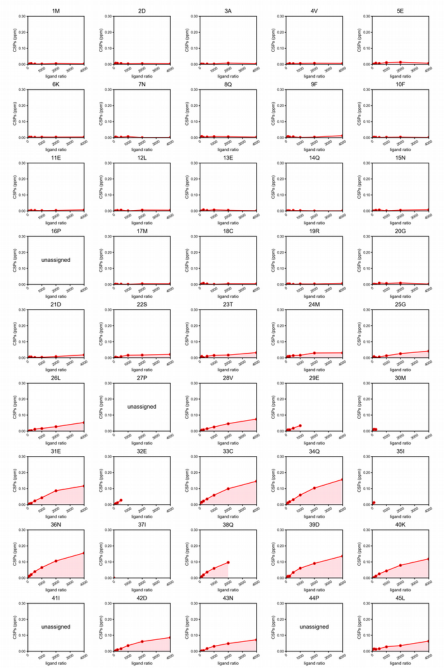

Figure 29: The full figure as generated by Farseer-NMR where a subplot is drawn for each residue.

Chemical Shift Scatter Plot

One of the most innovative plots of Farseer-NMR is the Chemical Shift Scatter plot: it translates to a plot the chemical shift evolution in two observed dimensions (generally ^{1}H and ^{15}N) for each residue, separately. The generated figure is amassed into one subplot for each residue in an *MxN* matrix. It is designed to fit a page width under the *Supporting Information* of a scientific manuscript. Individual plots can be cropped externally and used in specific figures of the main article body (Figures 30 and 31).

- all text and labels are customizable (font type, size and style)
- customisable colours:
  - colour of gradients
  - colour of shapes
  - colour of missing data points (' residues')
- customisable points styles: list of ordered shapes or colour gradient circle.
- identification of unassigned and missing residues
- externally configurable rules (default to 0.01 ppm) that is centred at the origin

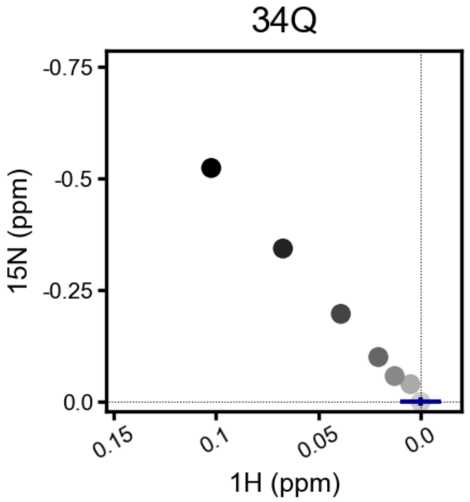

Figure 30: A subplot template representing the chemical shift perturbation of a specific peak where the reference is centred at 0.0.

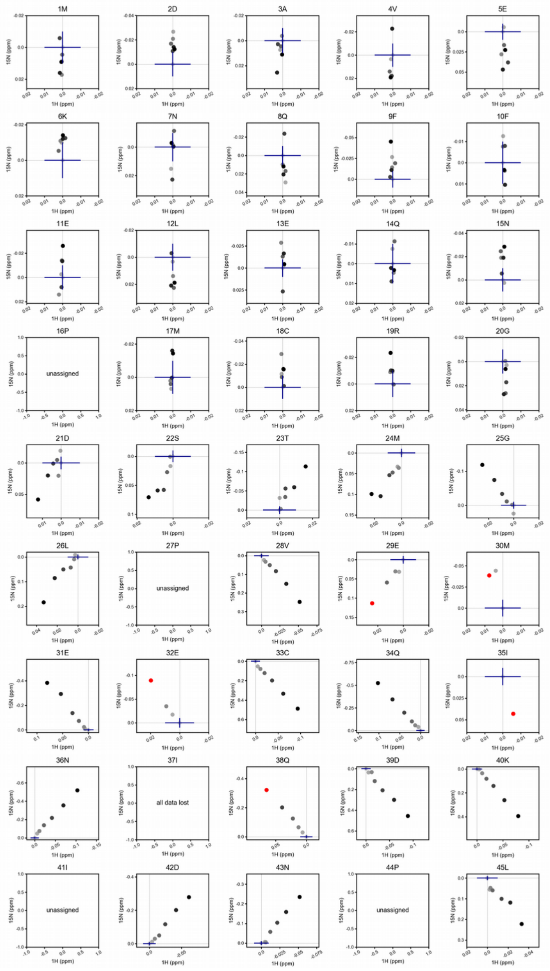

Figure 31: The full figure as generated by Farseer-NMR where a subplot is drawn for each residue.

Chemical Shift Scatter Flower Plot

Following the idea of the Chemical Shift Scatter Plot, the *Chemical Shift Scatter Flower* plot amasses all that information in a single plot. The spread of the chemical shifts away from the centre resemble a flower's petals, allowing easy discrimination of affected residues and grouping of them according to their changing nature(s) (Figure 32).

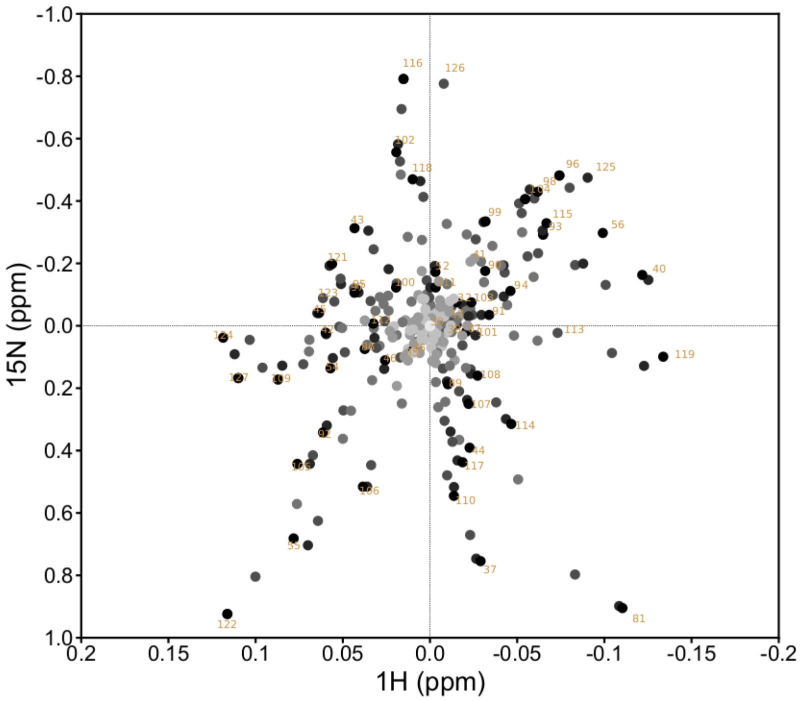

Figure 32: The Chemical Shift Scatter Flower plot.

The ΔPRE map

The ΔPRE map is generated only for the Comparative/Stacking analysis and for the Cz/ (Z axis) dataset, (further reading). It represents the ΔPRE values for the reference experiment and a smoothed line resulting from the Gaussian filter, as explained in Arbesú et al. 2017, overlapped with the same analysis for the datapoint under observation. A gradient colour can be applied along the series to represent the progression of the data. A tag pin is placed at the paramagnetic tag position for representation. User-defined regions or residues can be, shaded or highlighted, respectively.

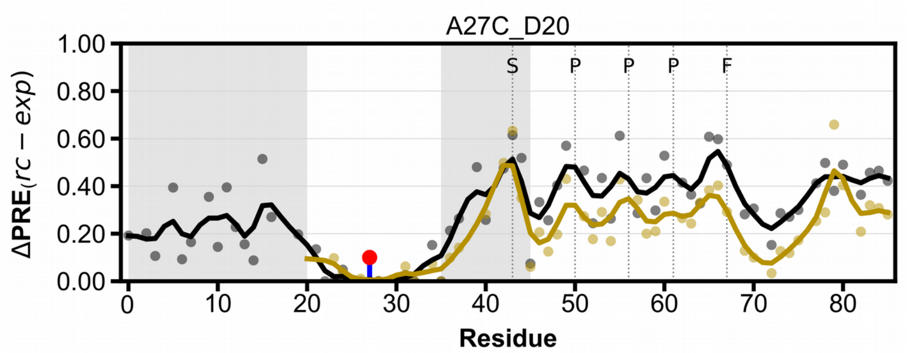

Figure 33: ΔPRE map. In black the values for the reference experiment. In gold the values for A27C_D20 construct. Regions 0-20 (Met1 is considered Met0 in this construct) and 35-45 are shaded to grey and residues 43, 50, 56, 61 and 67 are highlighted as examples. Data from Arbesú et al. 2017.

The ΔPRE heatmap

See here for a complete explanation on ΔPRE and how these results are stored. The ΔPRE heatmap represents the ΔPRE as a barcode giving a colour for each residue within a gradient where 1 is maximum relaxation enhancement and 0 is no enhancement (the upper and lower limits of this gradient can be configured). Results are better represented upon data smoothing with Gaussian filter.

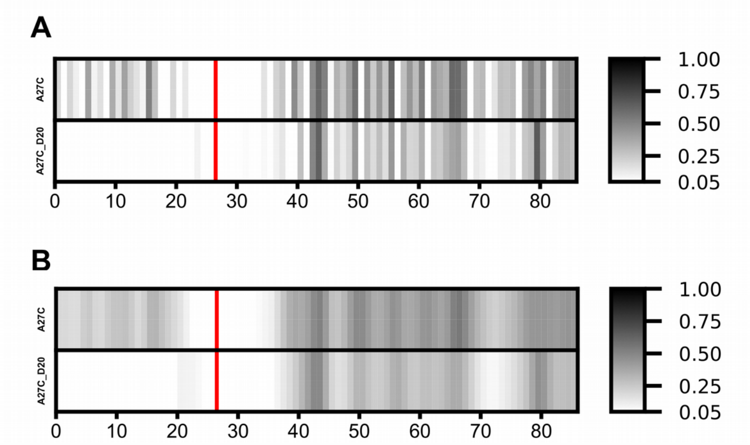

Figure 34: The ΔPRE heatmap templates. A) with raw data. B) with Gaussian smoothed data. Tag position if represented by a red line.

VII. Tutorials

This section contains different tutorial exercises that will help the user to get introduced to Farseer-NMR software and to its various applicabilities. The examples presented are inspired in real cases, however, NMR observables were modified or intentionally generated to better fit the educative purpose of the tutorials and, the original protein sequences were substituted by purely randomly generated sequences.

a) Tutorial 1 – Single variable datasets

Tutorial 1 describes the analysis of a single variable dataset, also termed **1D dataset**, it can be found in folder Documentation/Tutorial_Datasets/Tutorial_1. This section focuses on the analysis of a series of NMR peaklists that represents the evolution of the system as a function of a single experimental variable – the simplest case study. For example: investigating the binding event between proteinA and proteinB where proteinA is observed by ^{1}H-^{15}N HSQC NMR and the concentration of proteinB (unlabelled) is the independent variable. We deem this kind of dataset a 1D dataset because it spans a single dimension on the **Farseer-NMR Cube**.

Context

A reference ^{1}H-^{15}N HSQC NMR experiment was measured for proteinA at 200µM, representing the ligand free NMR fingerprint of the protein. Consecutively, six additional ^{1}H-^{15}N HSQC NMR experiments were acquired for proteinA in the presence of different concentrations of proteinB, ranging: 25, 50, 100, 200, 400 and 500µM. HSQC experiments were then analysed by the user's favourite NMR Analysis Suite and peaklist was exported for each of the spectra, containing information on the peaks assignment, chemical shift, intensities, user notes, etc. Peaklists were exported in convenient table formatted (.csv) files.

Aim

Describe the binding event between proteinA and proteinB:

- Identification of unassigned peaks
- Identification of missing peaks along the binding series
- Calculation of Chemical Shift Perturbations (CSPs)
- CSP representation for the whole proteinA along the series
- CSP representations for each single proteinA's residue along the series

How to

In Documentation/Tutorial_Datasets/Tutorial_1/pkls/ you will find the series of peaklists .csv files along with a proteinA.fasta file which contains the complete proteinA construct's sequence. A .fasta file needs to be provided for identification of unassigned residues.

1. Launch Farseer-NMR.
2. Remember that at any time of the configuration process you can **Save Configuration** and/or **Load Configuration** to import a previously saved state (further reading).
3. The first step is to select the folder where all the peaklists are stored.
    1. Go to **Settings Tab**, browse the **Peaklist Folder** field and select Documentation/Tutorial_Datasets/Tutorial_1/pkls.
    2. In the **Peaklist Selection Tab** the peaklist file names appear in the **Side bar**.
    3. You can also Drag&Drop individual files to the **Side bar**.
4. Create an experiment tree that defines the experimental variables investigated, thus describing the dataset to be analysed. Farseer-NMR requires the user to define always three different variables (Z, Y and X). In cases where less than three variables are analysed, simply define a single point in the unused axes.
    1. Create one single point in the Z axis and name it a temperature value, you can name it whatever but a temperature condition is a good practice to keep your data organized.

2. Create one single point in the Y axis and name it proteinA.
3. Create seven different points in the X axis and name them accordingly to the concentration range.

5. Click **Setup Experimental Series** (Figure 35).
6. Drag and Drop the peaklist names from the **Side bar** to the "Drop peaklist here" corresponding places in the **Experimental Tree** (Figure 36).
7. On the **Settings Tab** configure the following options:
8. The option **Analysing Along X axis** comes activated by default, in this tutorial there is no need to change this option.
9. Check **Apply FASTA?** And select the .fasta file for proteinA. As described in the Documentation manual, fasta files can only be selected for data points in the Y axis, that is why the Y axis codes for the protein construct observed (Figure 37 - 1).
10. Activate the NMR parameter that you wish to calculate, in this case, **chemical shift perturbations** are already activated be default, you can select additionally the **F1 data** (^{1}H) and **F2 data** (^{15}N) (Figure 37 - 2).
11. You can define a general α value for CSP calculation (Figure 37 – 3) or specify it separately for each protein residue type (Figure 38), default values are 0.14 for all but Glycine that is set to 0.2 (Williamson 2013).
12. Select the desired plotting templates. We recommend **Extended Bar**, **Compacted Bar** and **Residue Evolution** and **CS Scatter** (Figure 37 – 4 and 5)
    1. You can configure plotting details in the corresponding menus.
    2. To properly configure the **Residue Evolution Plot:**
        1. go to **General Evolution Settings** and in **Titration X values** define the concentration range or proteinB (Figure 39) writing the following: 0,25,50,100,200,400,500
        2. go to **Residue Evolution Settings** and activate the **Use User Defined X Values?** flag (Figure 40).
        3. **Optional:** activate the Fitting routine in **General Evolution Settings → Fit Parameter Evolution** so that the CSP values is fit to the Hill Equation for demonstration purposes only. In this way you can visualize how Farseer-NMR would perform data fitting to any implemented equation.
13. **Run Farseer-NMR**! Please recall that, to avoid data overwrite the calculation won't proceed if there is already a Backbone/ and/or a spectra/ folder in the **Calculation Output Folder**, these folder should be moved or deleted priorly. All information regarding the run will be printed to the terminal window and stored in the farseer_log.md file.

Results

A spectra/ folder and a Backbone/ folder are created inside the **Calculation Output Folder** defined in the run settings, please read the Results folder Hierarchy section for more information on how results are organized.

Inside the Backbone/ folder there is the Calculations/ folder with the following subfolder structure:

```
Backbone/
        Calculations/
                along_x/
                        00_298K/
                                00_proteinA/
```

where along_x/ contains the series extracted along the X axis of the Farseer-NMR Cube. In this Tutorial the Farseer-NMR cube contains only one datapoint along the Y and Z axes and therefore there are no series to be extracted along those dimensions; this was also not defined in the settings anyway. 00_298K/ and 00_proteinA/ folders are the Z and Y fixed data points, respectively, for the X series generated, that, in this case, is only one series. Inside 00_proteinA/ folder, you will find the folders that organize the Farseer-NMR output for the analysed series. Further reading on these folders and how are they organized is available. You will find this organization for each and every single series that is analysed by Farseer-NMR.

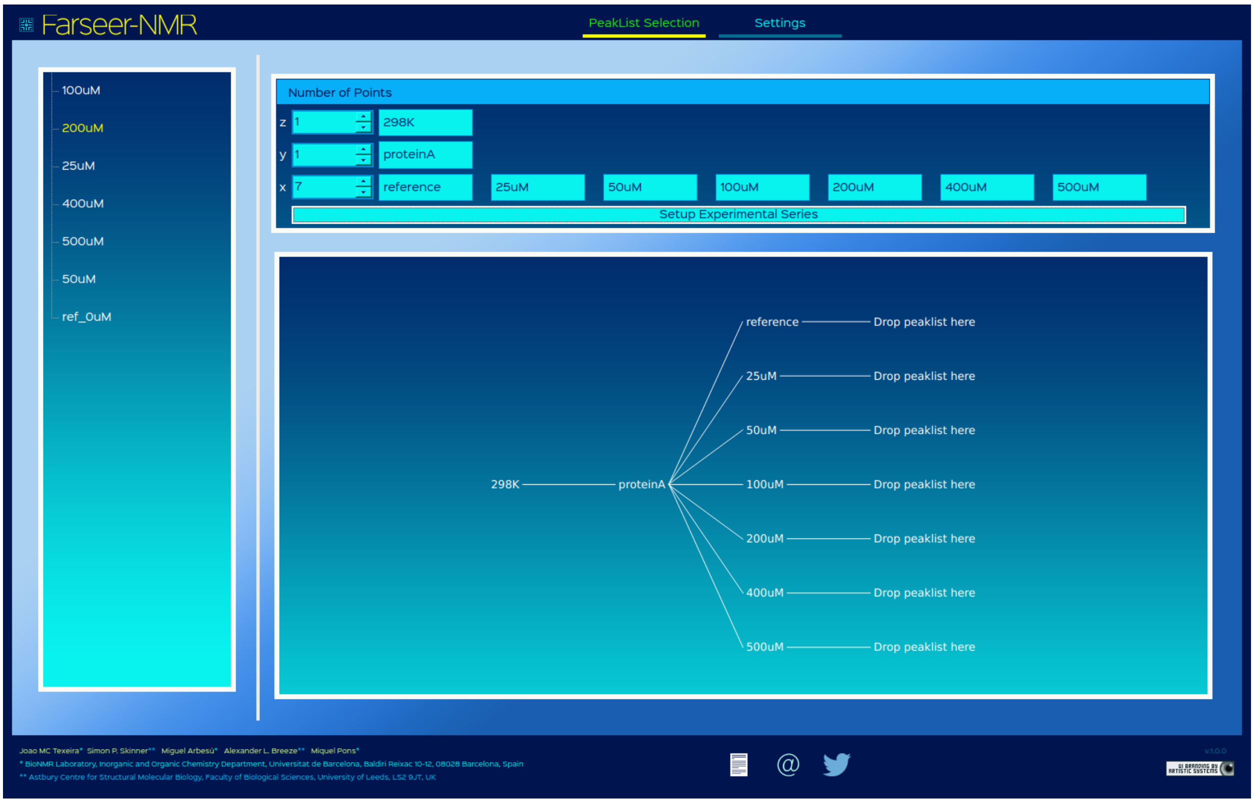

Figure 35: Experimental Tree defined and peaklists loaded to Sidebar – Tutorial 1.

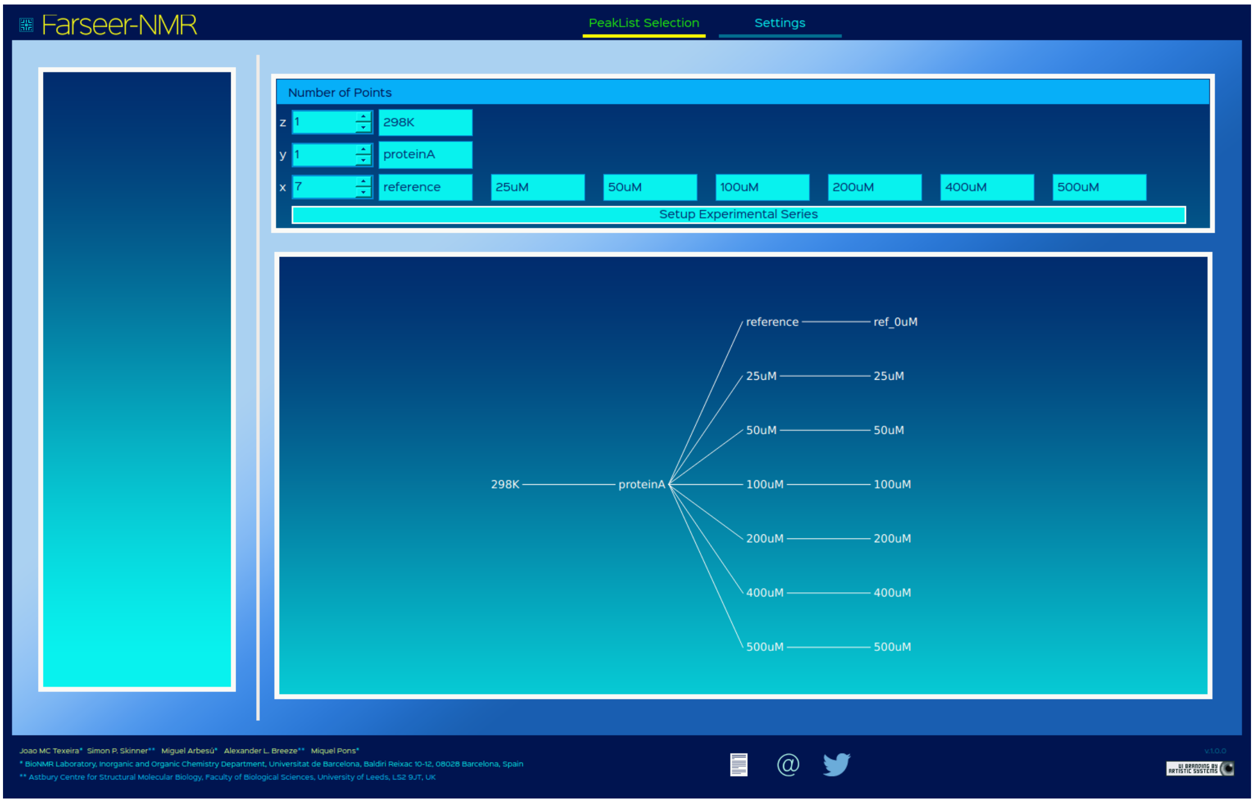

Figure 36: Experimental Tree populated – Tutorial 1.

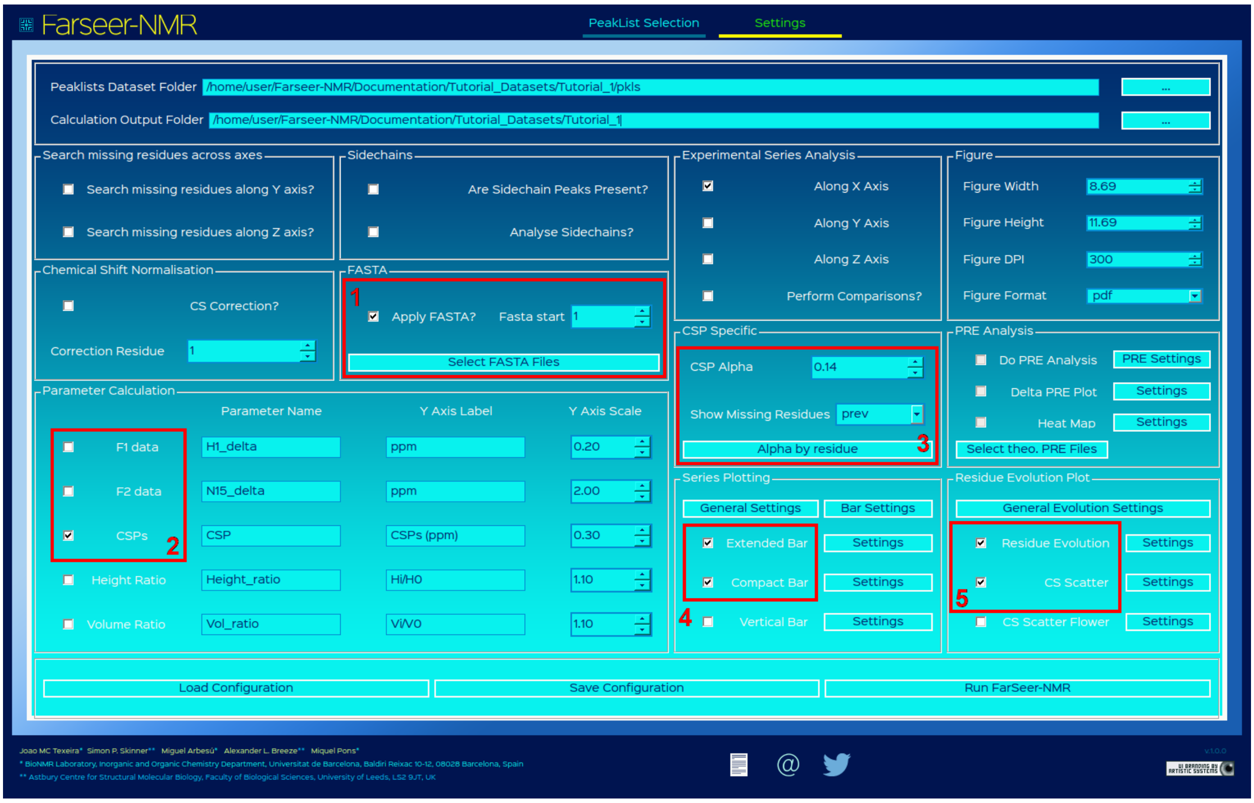

Figure 37: Configurations in Settings tab for Tutorial 1.

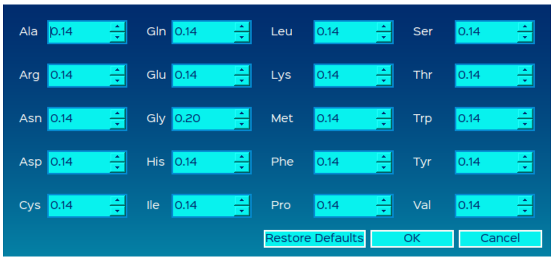

Figure 38: Configure individual CSP alpha values – Tutorial 1.

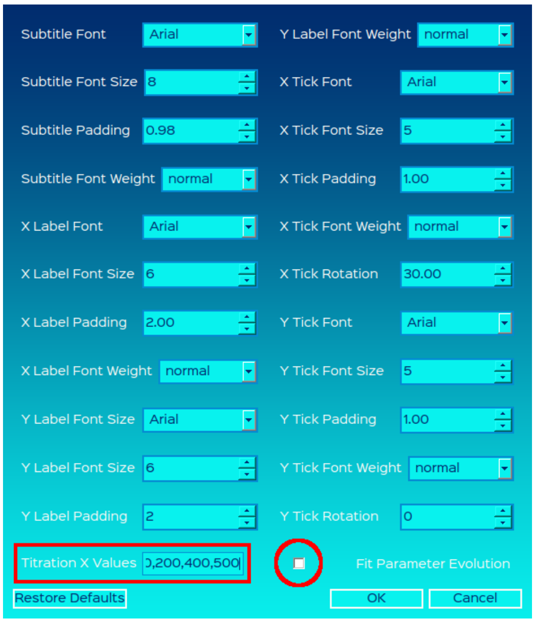

Figure 39: Configure concentration values for Residue Evolution Plot – Tutorial 1.

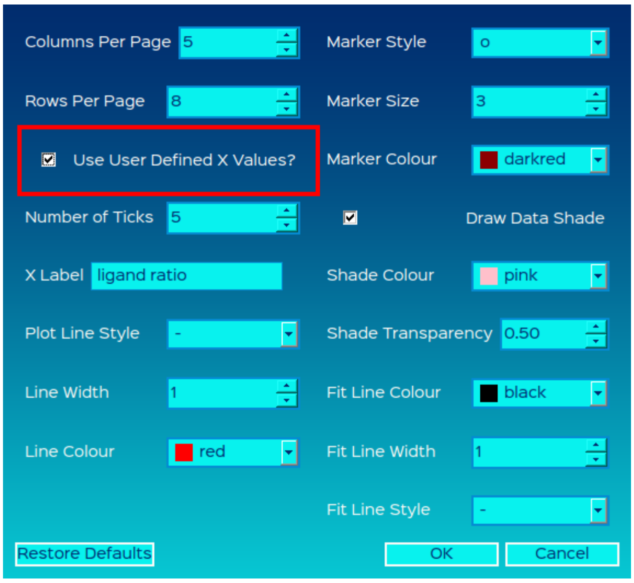

Figure 40: Configure Residue Evolution Plot – Tutorial 1.

b) Tutorial 2 – Analysing 2D Datasets

Tutorial 2 describes the analysis of a dataset containing peaklists that correlate two different variables, protein:protein ratio gradient and different protein constructs, also termed **2D dataset;** it can be found in folder Documentation/Tutorial_Datasets/Tutorial_2. In this case, we will perform:

- Analysis along the Farseer-NMR Cube's X axis, to:
  - evaluate the response of the system to increasing concentration of an unlabelled partner.
  - Two distinct mutants are evaluated.
- Analysis along the Y axis, to:
  - evaluate the experimental differences between the two mutants for the different partner concentration ratio.
- A Comparative/Stacking analysis between all the series evaluated.

We advise you to try Tutorial 1 prior to this one because the most basic things regarding Farseer-NMR calculations are explained there.

Context

ProteinA interacts with ProteinH weakly provoking small chemical shift perturbations. It is suspected that residue 39 has an active role in the interaction process. To understand the role of residue 39, two mutants were prepared, Ala39 and Glu39. The two ProteinA mutants were observed by ^{1}H-^{15}N HSQC NMR in the free state and in the presence of ProteinH at two different concentration ratios, 1:1 and 1:2. Because the interaction is weak, to facilitate the identification of the binding event, proteinH was functionalized with a paramagnetic MTSL tag, so that interaction between the two proteins results in decreasing of proteinA's signal intensity due to proximity with the paramagnetic MTSL tag. *This is example is not intended to demonstrate a deep analysis of paramagnetic data, we wish to demonstrate here a simple case. A further extension to different sets of paramagnetic data analysis see is provided in Tutorial 3. This example can be done equally well by considering CSPs, but we have intentionally used a paramagnetic case.*

Aim

The aims described for Tutorial 1 also apply. Describe the binding between the two *proteinA* mutants and *proteinH* and compare the differences between the two cases.

How to

1. Launch Farseer-NMR.
2. Remember that at any time of the configuration process you can **Save Configuration** and/or **Load Configuration** to import a previously saved state (read further).
3. The first step is to select the folder where all the peaklists are stored.
   1. Go to **Settings Tab**, browse the **Peaklist Folder** field and select Documentation/Tutorial_Datasets/Tutorial_2/pkls.
   2. In the **Peaklist Selection Tab** the peaklist file names appear in the **Side bar**.
   3. You can also Drag&Drop individual files to the **Side bar**.
4. Create an experiment tree that defines the experimental variables investigated, thus describing the dataset to be analysed. Farseer-NMR requires the user to define always three different

variables (Z, Y and X). In cases where less than three variables are analysed, simply define a single point in the non used axes.

1. Create one single point in the Z axis and name it a temperature value, you can name it whatever but a temperature condition is a good practice to keep your data organized.
2. Create two points in the Y axis and name it MutA and MutE.
3. Create three different points in the X axis and name them accordingly to the concentration range.

5. Click **Setup Experimental Series**, Drag and Drop the peaklist names from the **Side bar** to the "Drop peaklist here" corresponding places in the **Experimental Tree** (Figure 41).
6. Select the **Calculation Output Folder** in the **Settings tab**.
7. On the **Settings Tab** configure the following options:
8. The option **Analysing Along X axis** comes activated by default. On this example activate also **Analysis Along Y axis** and **Perform Comparisons?**, as we do want now to analyse in both X and Y dimensions as well as stack the obtained results along different axis to allow easier visualization.
9. Activate **Height Ratio** in **Parameter Calculation**. You can also keep the CSPs calculation flag activated. There is no information regarding the Volume in this dataset.
10. Activate the **Apply FASTA?** Flag and select the .fasta file for the two different Y data points (Figure 42). Because we are observing two different protein constructs (mutants) Farseer-NMR requires two different .fasta files to be input. Further reading: information on different constructs must be configured along the Y axis as described in the **Axes Restriction section** of the **Documentation Manual**.
11. Activate **Compact Bar Plot** and **Residue Evolution Plot**.
    1. Change the specific plotting settings according to your desires.
    2. In **Residue Evolution Plotting Settings** do not activate the **Use User Defined X Values** because in this case we are not considering absolute concentrations, rather only concentration ratios.
12. **Run Farseer-NMR**! Please recall that, to avoid data overwrite the calculation won't proceed if there is already a Backbone/ and/or a spectra/ folder in the **Calculation Output Folder**, these folder should be moved or deleted priorly. All information regarding the run will be printed to the terminal window and stored in the farseer_log.md file.

Results

spectra/ and Backbone/ folders are created inside the **Calculation Output Folder** defined in the run settings, please read on the Results folder Hierarchy section for more information on how results are organized.

Inside the Backbone/ folder there are the Calculations/ and Comparisons/ folders with the following subfolder structure (see next page).

Inside Calculations/ you find the series that have been extract from the Farseer-NMR Cube and analysed following to the analysis routines. There are two sets of series, those generated along the X axis, stored inside along_x/ folder, and those generated along the Y axis, stored inside the along_y/ folder. Inside those folder you will find the calculation output organized according to what described in a previous section.

The Comparisons/ folder contains the results obtained from evaluating along the X and Y axes, Cx/ and Cy/ subfolders, respectively, staked along the other dimensions, along_y/ and along_x/, respectively for this case. Please read carefully the corresponding section Comparative/Stacking analysis to understand how theses results are organized.

```
Backbone/
       Calculations/
              along_x/
                     00_298K/
                            00_MutE/

                            01_MutA/

              along_y/

                     00_reference/

                            00_298K/

                     01_1eq/

                            00_298K/

                     02_2eq/

                            00_298K/

       Comparisons/

              Cx/

                     along_y/

                            00_reference/

                                   00_298K/

                            01_1eq/

                                   00_298K/

                            02_2eq/

              Cy/

                     along_x/

                            00_298K/

                                   00_MutE/

                                   01_MutA/
```

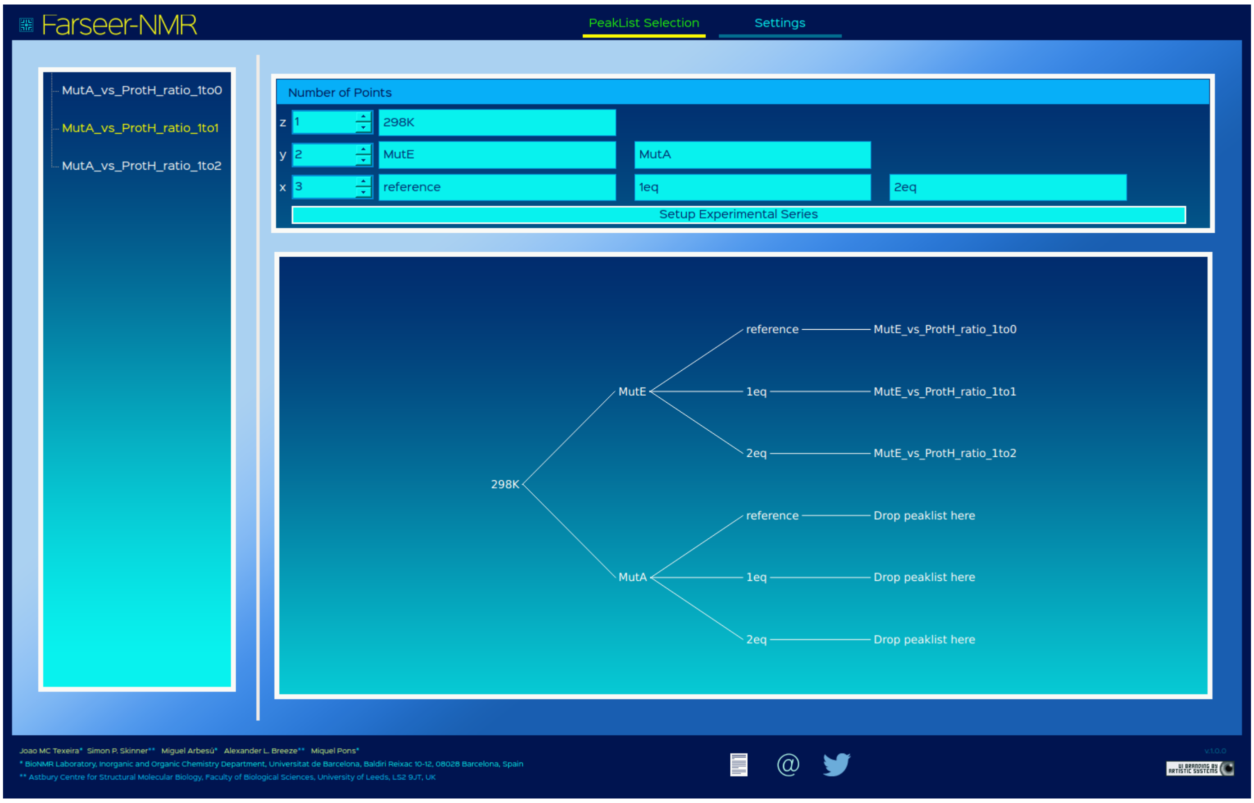

Figure 41: Setting up the Experimental Tree – Tutorial 2.

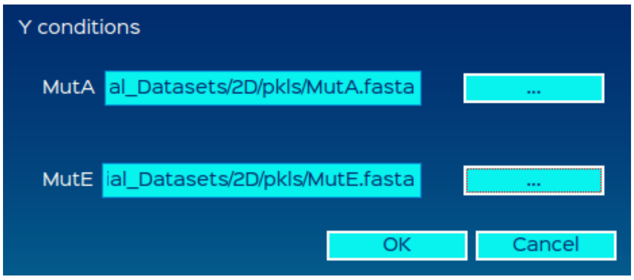

Figure 42: Loading FASTA files – Tutorial 2.

c) Tutorial 3 – paramagnetic NMR analysis

Tutorial 3 explores the analysis of paramagnetic NMR data for four different experimental setups that have been measured in both diamagnetic and paramagnetic conditions. Please complete Tutorial 1 and Tutorial 2 before attempting this exercise. This example uses axes Y and Z but it can be easily expanded to also include the X axis. In this example, we will also make of use of two routines that are restricted to certain axis, which are analysis of different constructs (Y axis) and paramagnetic data (Z axis).

Context

An intrinsically disordered protein of 80 residues was mutated in position A2C and A27C, the engineered cysteine was functionalized with a MTSL paramagnetic tag with the intention of inspecting the protein's intramolecular interactions. The first twenty residues are suspected to have an important role in these intramolecular interactions, therefore, two truncated constructs were generated from the mutant A27C: D10 and D20 where the first ten and twenty residues were removed, respectively. Two ^{1}H-^{15}H NMR HSQC experiments were measured for each construct, a paramagnetic experiment followed by a diamagnetic reference after treatment with 5-equivalents of ascorbic acid. All experiments were acquired at 278K, which is the temperature at which peaks have highest intensity in this case. In order to understand how the dynamics of this intrinsically disordered protein deviate from a random coil model, the theoretical paramagnetic relaxation enhancements (PRE) were calculated with Flexible Meccano and used in the Farseer-NMR analysis.

Aim

Analyse how the intramolecular interactions change with the change in construct nature by representing the peak intensity ratios between the paramagnetic and the diamagnetic (reference) series. Compare those with the theoretical values that consider a random coil model. Additionally, apply the DPRE analysis.

How To

1. Launch Farseer-NMR.
2. Remember that at any time of the configuration process you can **Save Configuration** and/or **Load Configuration** to import a previously saved state (read further).
3. The first step is to select the folder where all the peaklists are stored.
    1. Go to **Settings Tab**, browse the **Peaklist Folder** field and select Documentation/Tutorial_Datasets/Tutorial_3/pkls.
    2. In the **Peaklist Selection Tab** the peaklist file names appear in the **Side bar**.
    3. You can also Drag&Drop individual files to the **Side bar**.
4. Create an experiment tree that defines the experimental variables investigated, thus describing the dataset to be analysed. Farseer-NMR requires the user to define always three different variables (z, y and x). In cases where less than three variables are analysed, simply define a single point in the non used axes (in this case X is a single data point axis) (Figure 43).
    1. Paramagnetic analysis must be configured along the Z axis. Setup two datapoints along Z: "diamagnetic" and "paramagnetic".
    2. Input information on the constructs in the Y axis: create four datapoints, "A2C", A27C", "A27C_D10" and "A27C_D20".
    3. Create a single point in the X axis and name it equally to the experimental temperature, "278K".

5. Configure the Farseer-NMR Run according to the following (Figure 44):
6. In the **Settings tab**, activate analysis over all axes plus comparisons.
7. Activate **Search missing residues along Z axis?** (read further).
8. Select the corresponding FASTA files for each Y axis datapoint. In this case two different .fasta files are required, that corresponding to the A2C mutant and other for the A27C mutant and constructs (Figure 45). Here we use the same .fasta for the truncated constructs because we want that those truncated residues are identified in the plots (as unassigned residues) and because we want to compare the different constructs and for that computational analysis requires that all datasets have the same number of rows (residues) so that data tables (peaklists) can be compared.
9. Activate the calculation of Height ratios under **Parameter Calculation**.
10. When activating **Height** or **Volume Ratios** the **PRE Analysis** box becomes active allowing the user to select ΔPRE analysis. Activate the three PRE Analysis checkboxes. This activates the analysis itself (**Do DPRE Analysis**) and the generation of the DPRE map and the DPRE heatmap.
11. The DPRE Analysis requires that information on theoretical PRE profiles are also input. A file containing the theoretical PRE should be selected for each Y axis data point, this is done in the same way as for the .fasta files. Use the menu **Select theo. PRE Files** to select the corresponding .pre files (Figure 46).
12. Activate the **Compacted Bar Plot** representation (is the best suited for this example).
13. **Run Farseer-NMR**! Please recall that, to avoid data overwrite the calculation won't proceed if there is already a Backbone/ and/or a spectra/ folder in the **Calculation Output Folder**, these folder should be moved or deleted priorly. All information regarding the run will be printed to the terminal window and stored in the farseer_log.md file.

Results

Farseer-NMR analysis along the Y axis and the Z axis occur as described in the previous examples (Tutorial 1 and Tutorial 2 results sections) for X and Y. You can find those in Backbone/Calculations/along_y/ and along_z/.

Before continuing, please consider reading the PRE and DPRE explanatory section.

However, there is an addition to the analysis along the Z axis (along_z/ folder). The theoretical PRE profiles appear for the Height and/or Volume ratio parameters in the subplots for the paramagnetic data point (TableAndPlots/ folder). Because the first subplot is always evaluated against itself, the diamagnetic subplot of Height ratio yields 1 for every residue (Figure 47).

The folder PRE_Analysis/ (under along_z/) contains the plots and representations that are specific for this analysis. Read further on the Delta PRE analysis.

In the example presented in this tutorial, we can reap considerable benefits from the Comparison/Stacking routines. For example, what if we wanted to observe, in a single figure, the changes in PRE as a function of the protein construct. You can access this plot under:

Backbone/Comparisons/Cz/along_y/00_278K/01_para/TablesAndPlots/Height_ratio/

The stacking routine gathers all plots generated along_z for the different Y datapoints in a single figure. The same could be applied along the X axis, for example to visualize the evolution of PRE profiles as a function of a ligand concentration.

Following on from the above, of the PRE Analysis routines, the DPRE Map is a special case. This map is a representation of the ΔPRE profiles along the evolution of another variable and, therefore, it can only be represented in a Stacking analysis. To find this plot go to:

Backbone/Comparisons/Cz/along_y/00_278K/01_para/TablesAndPlots/PRE_analysis/

it won't show for the diamagnetic (00_dia/) series for obvious reasons.

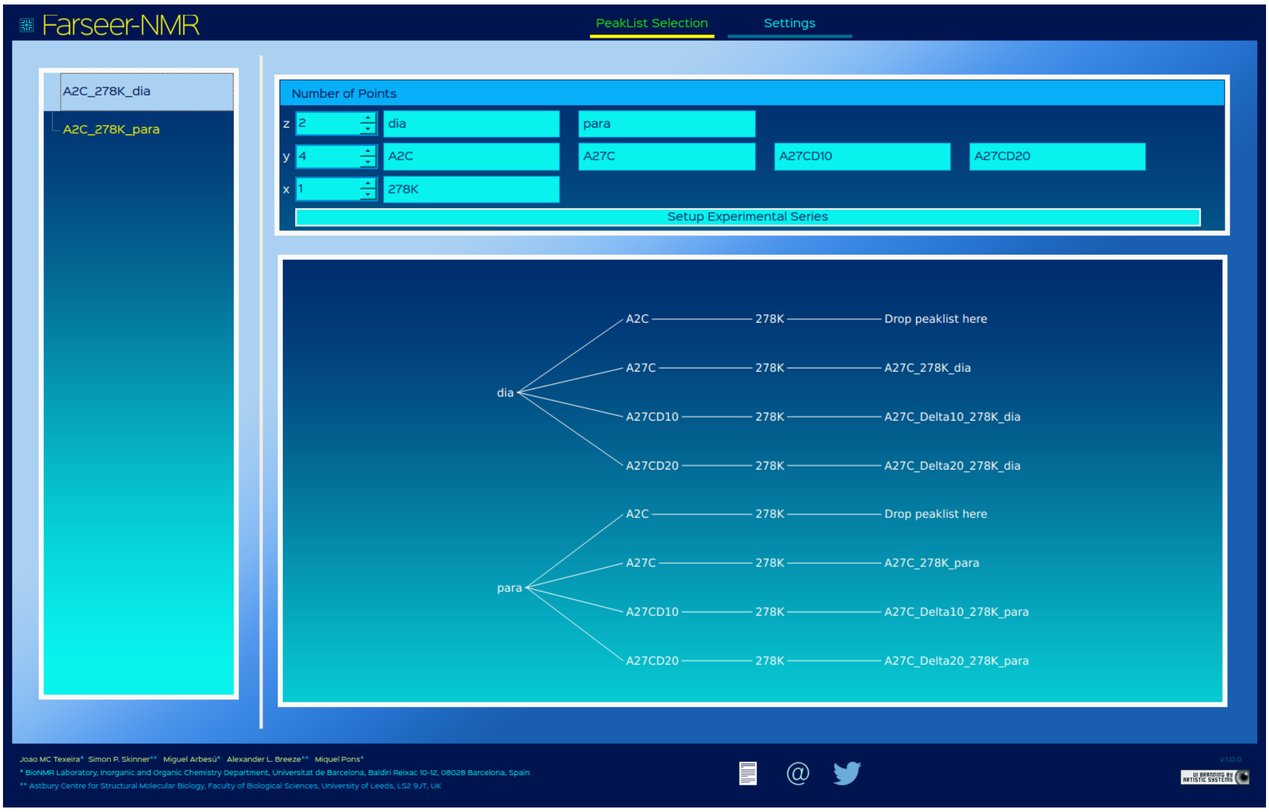

Figure 43: Populating the Experimental Series Tree – Tutorial 3.

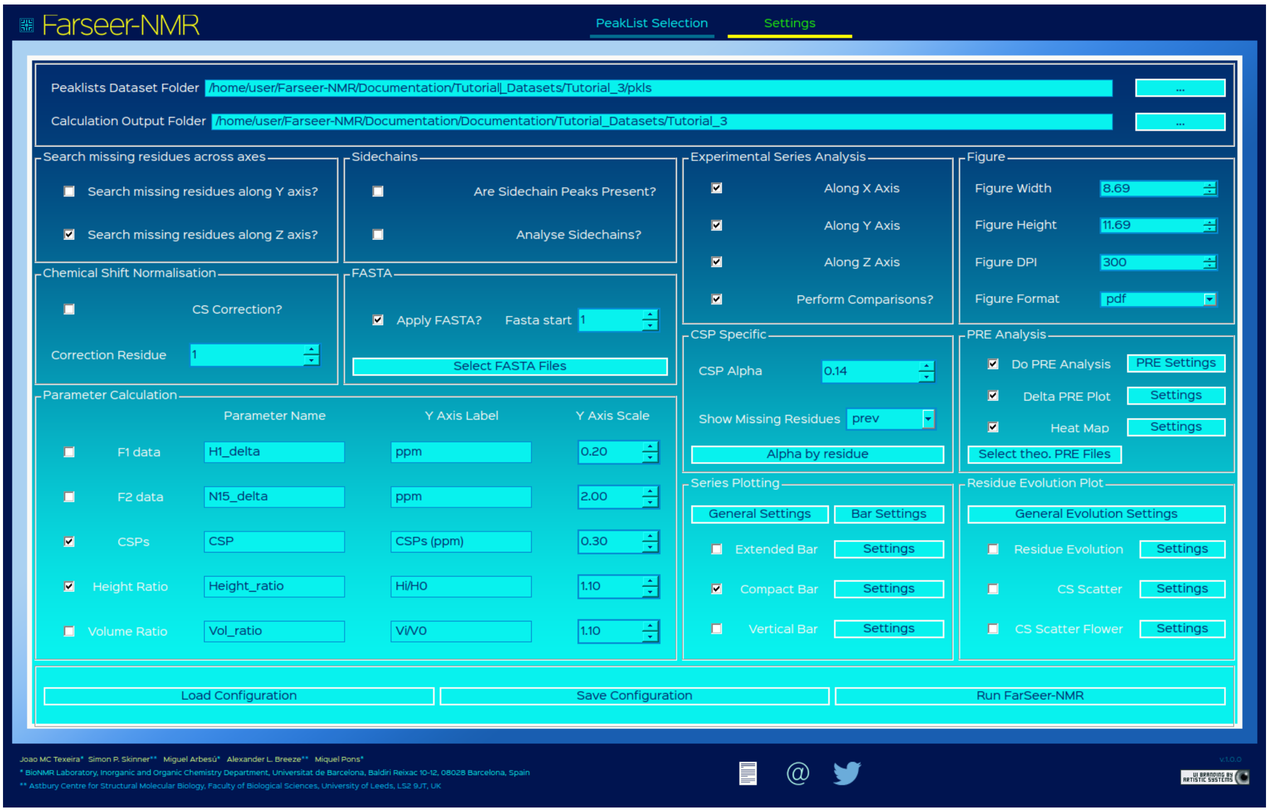

Figure 44: Settings configuration for Tutorial 3.

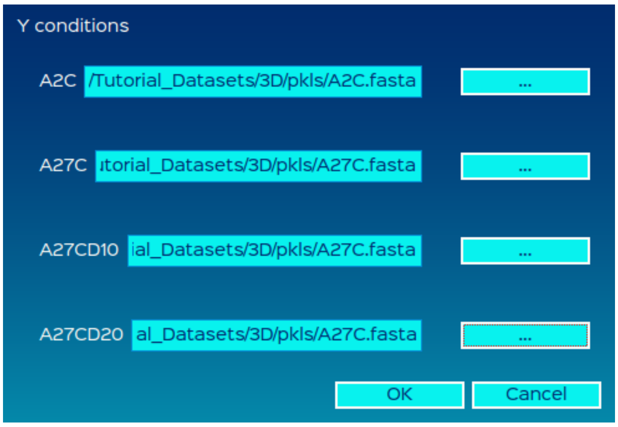

Figure 45: Loading .fasta files for Y axis – Tutorial 3.

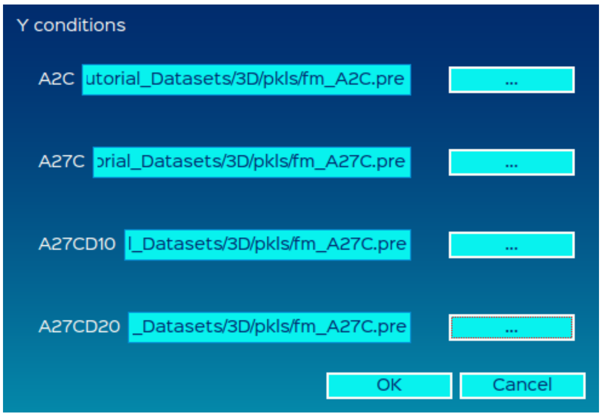

Figure 46: Loading .pre files for Y axis – Tutorial 3.

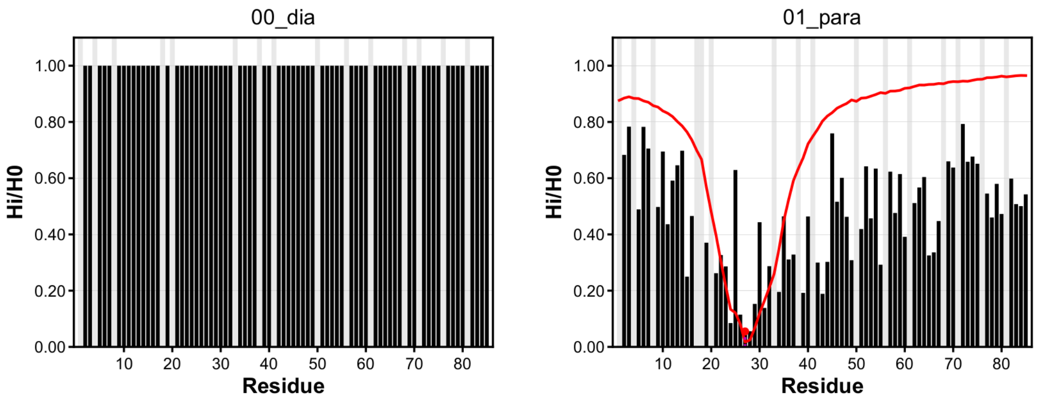

Figure 47: Compacted Bar plot for A27C (278K) along the Z axis. The datapoint corresponding to the paramagnetic data represents the input theoretical PRE profile. While the diamagnetic is a division over the self yielding 1.

THANK YOU
